# Gastroprotective Efficacy of North African Medicinal Plants: A Review on Their Therapeutic Potential for Peptic Ulcers

**DOI:** 10.1002/fsn3.4536

**Published:** 2024-10-22

**Authors:** Nezar Cherrada, Ahmed Elkhalifa Chemsa, Noura Gheraissa, Ibtissam Laib, Zakia Gueboudji, Mohamed EL‐Shazly, Abdelmalek Zaater, Asma Abid, Sherouk Hussein Sweilam, Talha Bin Emran, Sadok Nani, Bilal Benamor, Djilani Ghemam Amara, Ayomide Victor Atoki, Mohammed Messaoudi

**Affiliations:** ^1^ Faculty of Life and Natural Sciences, Department of Biology University of El Oued El‐Oued Algeria; ^2^ Laboratory of Biodiversity and Application of Biotechnology in Agriculture University of El Oued El‐Oued Algeria; ^3^ Faculty of Life and Natural Sciences, Department of Molecular and Cellular Biology University of El Oued El‐Oued Algeria; ^4^ Faculty of Nature and Life Sciences Abbes Laghrour University of Khenchela Khenchela Algeria; ^5^ Biotechnology, Water, Environment and Health Laboratory Abbes Laghrour University of Khenchela Khenchela Algeria; ^6^ Faculty of Pharmacy, Department of Pharmacognosy Ain Shams University Cairo Egypt; ^7^ Faculty of Life and Natural Sciences, Department of Agronomy University of El Oued El‐Oued Algeria; ^8^ Faculty of Mathematics and Matter Sciences University of Ouargla Ouargla Algeria; ^9^ Laboratory of Valorization and Promotion of Saharan Resources (VPRS) Ouargla Algeria; ^10^ College of Pharmacy, Department of Pharmacognosy Prince Sattam Bin Abdulaziz University Al‐Kharj Saudi Arabia; ^11^ Faculty of Pharmacy, Department of PharmacognosyCairo‐Suez Road Egyptian Russian University Badr City, Cairo Egypt; ^12^ Warren Alpert Medical School, Department of Pathology and Laboratory Medicine Brown University Providence Rhode Island USA; ^13^ Legorreta Cancer Center Brown University Providence Rhode Island USA; ^14^ Faculty of Allied Health Sciences, Department of Pharmacy Daffodil International University Dhaka Bangladesh; ^15^ Higher School of Saharan Agriculture‐El Oued El Oued Algeria; ^16^ Laboratory of Genetic, Biotechnology and Valorization of Bio‐Resources (LGBVB) University of Mohamed Khider Biskra Algeria; ^17^ Laboratory Biology, Environment, and Health University of El Oued El‐Oued Algeria; ^18^ Department of Biochemistry Kampala International University Ishaka Uganda; ^19^ Nuclear Research Centre of Birine Ain Oussera, Djelfa Algeria

**Keywords:** alternative therapies, gastroprotective effects, *Helicobacter pylori*, North African medicinal plants, peptic ulcer, phytochemical constituents

## Abstract

Peptic ulcer disease remains a prevalent gastrointestinal disorder worldwide. Current treatments often have limitations, sparking interest in alternative therapies from medicinal plants. This review examines the gastroprotective potential of 54 North African medicinal plants against peptic ulcers. An extensive literature search was conducted, focusing on plants with preclinical and clinical evidence of anti‐ulcer efficacy and documented use in North African traditional medicine. The review identified several promising plant species, such as licorice (*Glycyrrhiza glabra*), chamomile (*Matricaria chamomilla*), olive (*Olea europaea*), pomegranate (*Punica granatum*), Aloe vera, and black seed (*Nigella sativa*), along with their bioactive constituents, including flavonoids, tannins, and terpenoids. These compounds exhibit gastroprotective properties through multiple mechanisms, such as enhancing the gastric mucosal barrier, inhibiting acid secretion, displaying antioxidant and anti‐inflammatory effects, promoting ulcer healing, and combating *Helicobacter pylori* infection. The evidence presented includes in vitro assays, animal models, and some clinical studies. While many of the 53 plants reviewed demonstrated significant anti‐ulcer effects compared to standard drugs, further clinical research is needed to establish efficacy and safety in humans. The synergistic actions of phytochemical mixtures in medicinal plant extracts likely contribute to their therapeutic potential. This review highlights the role these North African medicinal plants may play in the prevention and treatment of peptic ulcers and identifies promising candidates for further research and development of evidence‐based botanical therapies.

## Graphical abstract

Peptic ulcer disease remains common globally, driving interest in alternative treatments. This review examines 53 North African medicinal plants, including licorice, chamomile, olive, pomegranate, Aloe vera, and black seed, for their anti‐ulcer effects. These plants contain compounds like flavonoids and tannins that protect the stomach lining, lower acid production, reduce inflammation, and combat *Helicobacter pylori*. Although promising, further clinical studies are needed to confirm their safety and effectiveness in humans.

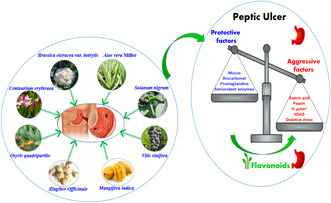

## 1 Introduction

Peptic ulcers are painful sores in the lining of the stomach or duodenum, affecting nearly 10% of the global population (Majumdar and Looi 2024). The primary etiological factors are *Helicobacter pylori* infection and frequent NSAID use, contributing to 60%–90% and 30%–50% of ulcer cases, respectively (Hooi et al. 2017; Malfertheiner et al. 2017). Additional factors include excessive alcohol consumption, smoking, stress, gastric hyperacidity, diet, genetics, and radiotherapy (Sostres, Gargallo, and Lanas 2013).

Symptoms range from mild discomfort to severe pain and include nausea, vomiting, bloating, and heartburn (Lanas and Chan 2017). Complications such as gastrointestinal bleeding, perforation, and gastric outlet obstruction can arise if left untreated, increasing the risk of gastric cancer (Crew and Neugut 2006). Timely diagnosis and proper management are crucial.

**FIGURE 1:**
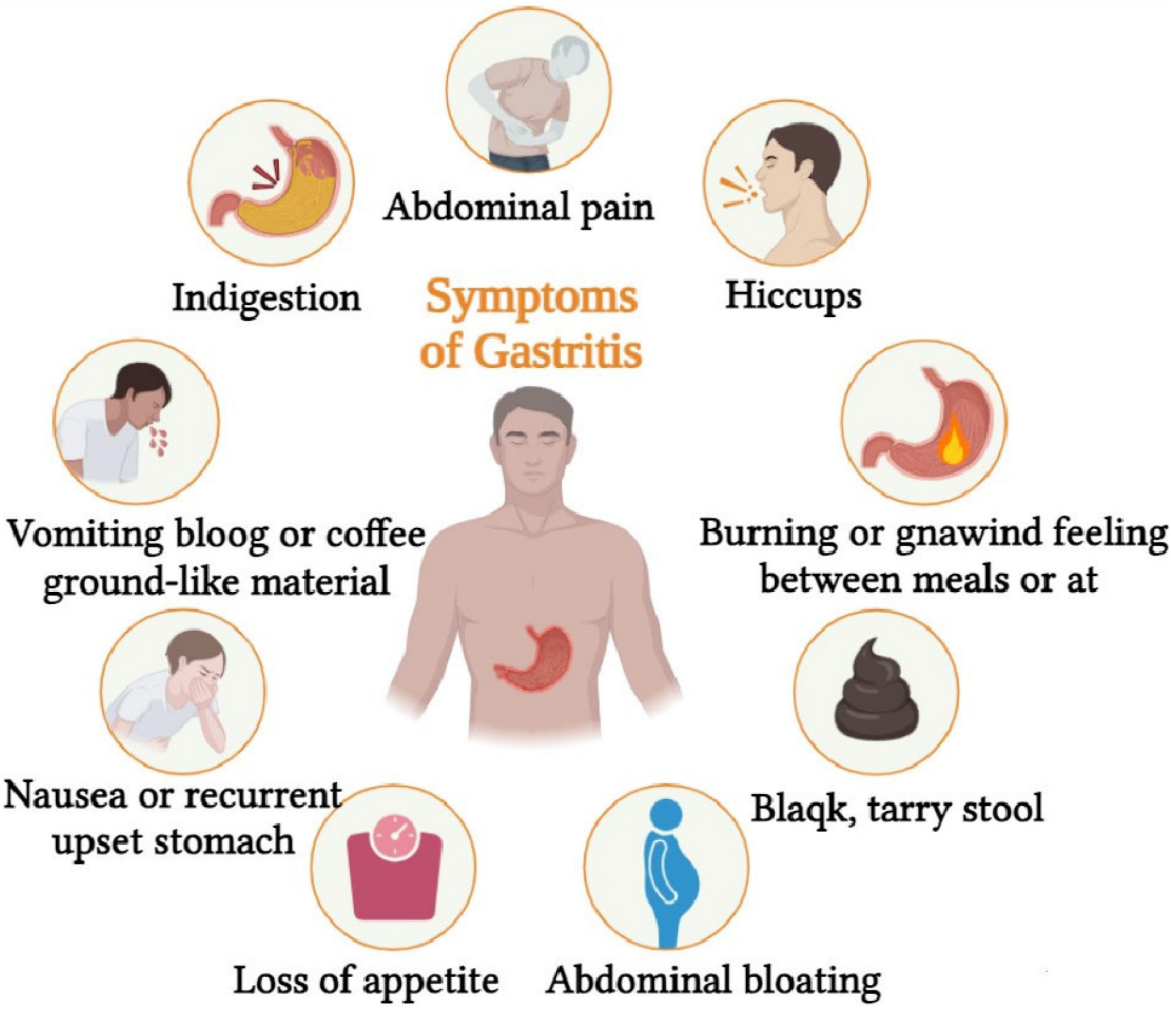
The symptom is classic for peptic ulcer.

Figure 1, under the heading “The symptom is classic for peptic ulcer,” presents a more comprehensive overview of the symptoms associated with peptic ulcers, detailing both the common and less frequent clinical manifestations.

Conventional treatments involve eradicating *H. pylori* and suppressing gastric acid secretion with antibiotics, PPIs, or H2 blockers (Malfertheiner et al. 2017). However, rising antibiotic resistance and long‐term side effects of PPIs, such as nutrient deficiencies and increased infection risks, highlight the need for alternative therapies (Fashner and Gitu 2015).

Traditional medicine offers a promising avenue, with North African medicinal plants gaining attention for their gastroprotective properties (Asnaashari, Dastmalchi, and Javadzadeh 2018). The rich biodiversity of North Africa, ranging from arid deserts to lush coastal regions, harbors numerous plants with a history of use in treating gastrointestinal disorders (Bouyahya et al. 2017; Ghanmi et al. 2014). Plants like olive leaf (*Olea europaea*), date palm (*Phoenix dactylifera*), and argan (*Argania spinosa*) have demonstrated anti‐ulcer effects through various mechanisms, including inhibition of gastric acid secretion and antioxidant activity (Rigacci and Stefani 2016; Silvan et al. 2021; Yasin, El‐Fawal, and Mousa 2015).

This review aims to summarize current scientific evidence on the gastroprotective efficacy of North African medicinal plants, examining their botanical sources, key phytochemical constituents, and mechanisms of action. By establishing the therapeutic potential of these traditional medicines, we hope to pave the way for novel botanical treatments for peptic ulcers.

## 2 Methods

A comprehensive literature search was conducted using PubMed, Scopus, ScienceDirect, Web of science, and Google Scholar, focusing on medicinal plants traditionally used in North Africa for treating gastrointestinal ulcers. Keywords included “anti‐ulcer plants,” “gastroprotective plants,” “peptic ulcer medicinal plants,” and “traditional anti‐ulcer herbs of North Africa.” References from relevant articles were also screened.

Inclusion criteria prioritized plants with robust evidence of gastroprotective properties demonstrated through preclinical in vivo and in vitro studies. We focused on botanicals with well‐established pharmacological validation and a documented history in North African ethnomedicine. Phytochemical studies were also reviewed to identify active compounds and their mechanisms of action.

This review synthesizes key findings from the literature, providing an overview of North African medicinal plants with potential anti‐ulcer effects, and aims to highlight their therapeutic viability as alternatives to conventional PUD treatments.

## 3 Medicinal Plants of North Africa

In the vast and varied landscapes of North Africa, traditional healing practices have flourished for centuries, deeply rooted in the rich biodiversity that thrives across the region. Indigenous communities, including the Tuareg, Bedouin, Amazigh (Berber), and Arab peoples, have cultivated an intimate relationship with the local flora, harnessing its medicinal properties to treat a wide array of ailments amid the challenges posed by the region's diverse environments (Alves‐Silva et al. 2017; Khalid et al. 2012; Miara et al. 2019). Spanning countries such as Morocco, Algeria, Tunisia, Libya, and Egypt, North Africa boasts a tapestry of ecosystems ranging from the arid expanses of the Sahara Desert to the fertile lands along the Mediterranean coast. Within this botanical mosaic, numerous plant families have emerged as prominent sources of medicinal remedies, including Asteraceae, Fabaceae, Apiaceae, Lamiaceae, Brassicaceae, Solanaceae, Apocynaceae, and Zygophyllaceae, among others (Benarba et al. 2015). The use of medicinal plants in North Africa is deeply intertwined with cultural traditions and indigenous knowledge systems. Passed down through generations, this wealth of botanical wisdom has enabled communities to address a wide range of health concerns, including gastrointestinal disorders, which have historically been prevalent in the region. The traditional healers of North Africa, often referred to as herbalists or healers, possess a deep understanding of the therapeutic properties of local plants and employ a holistic approach to healing that considers the interconnectedness of body, mind, and environment (Dinat, Orchard, and Van Vuuren 2023; Vale and Oleastro 2014). In the realm of gastrointestinal health, North African medicinal plants have garnered significant attention for their efficacy in treating conditions such as peptic ulcers, often caused by the bacterium *H. pylori*. This microorganism infects a substantial portion of the regional population and is implicated in the majority of duodenal and gastric ulcers. Research has identified several medicinal plants with potent anti‐*H. pylori* properties, including thyme (*Thymus vulgaris*), wild thyme (*Coridothymus capitatus*), rosemary (*Rosmarinus officinalis*), and turmeric (*Curcuma longa*) (Beladi Mousavi, Naghdifar, and Rafieian‐Kopaei 2016; Güvenir 2024; Reddy et al. 2023). These plants contain bioactive compounds that exhibit antibacterial activity against *H. pylori*, providing a natural alternative to conventional antibiotics. Furthermore, the berries of *Pistacia lentiscus* and Oleo gum resin from *Cistus ladaniferus* have demonstrated antibacterial effects against *H. pylori*, along with antioxidant properties that promote ulcer healing. Additionally, chamomile (*Matricaria chamomilla*) and licorice (*Glycyrrhiza glabra*) have been shown to exert gastroprotective effects by enhancing mucus production in the stomach lining or through anti‐secretory actions against gastric acid (Dinat, Orchard, and Van Vuuren 2023; Safavi, Shams‐Ardakani, and Foroumadi 2015).

However, the utilization of medicinal plants in North Africa is not without its challenges. Overexploitation and unsustainable harvesting practices threaten the existence of some valuable species, prompting calls for conservation efforts and sustainable management practices (Jamila and Mostafa 2014). Moreover, the lack of standardized dosages and potential interactions with conventional medications necessitate further research and clinical trials to validate their safety and efficacy in clinical settings (Naceiri Mrabti et al. 2021).

The table of the North African medicinal plants (Table 1) indicates a significant prominence of families such as Fabaceae, Asteraceae, and Lamiaceae, which are frequently utilized for their gastroprotective properties. Notable compounds including flavonoids (e.g., quercetin and kaempferol), tannins, alkaloids, and essential oils are commonly identified, and recognized for their anti‐inflammatory, antioxidant, and antimicrobial effects, all contributing to the treatment of peptic ulcers. The most utilized plant parts are the aerial parts, leaves, and roots, reflecting their high concentration of bioactive compounds.

**TABLE 1:** Investigated the peptic ulcers medicinal plants of North Africa and identified possible bioactive compound.

Scientific name	Family	Common names	Part(s)	Compound(s) present/isolated	Reference
*Abelmoschus esculentus*	Malvaceae	Okra or lady's finger	Aerial parts	Flavonoids (quercetin), carotenoids, alkaloids, saponins and tannins, starch, glycosides, and mucilage	Altaf et al. (2023); Naim et al. (2015)
*Acacia Senegal*	Fabaceae	Gum Arabic	The bark and roots	Ferulic acid, chlorogenic acid, quercetin, arabinogalactan, and polysaccharides	Khedr (2017); Mahamoud Saleh, El Sayed El Sahar, and Elnadi (2021)
*Aegle marmelos*	Rutaceae	Bael	Leaves and fruit	Essential oil, alkaloids, carbohydrates, glycosides, flavonoids, tannins, coumarins, sterols, and triterpenoid	Abdallah, Salem, and El‐Salam (2017); Ibrahim et al. (2015)
*Aerva persica*	Amaranthaceae	Cotton	Roots	Aervin, 3‐hydroxy‐4 methoxy benzaldehyde, ursolic acid, and (E)‐N‐(4‐hydroxy‐3‐methoxyphenethyl)‐3‐(4‐hydroxy‐3‐ethoxyphenyl) acryl amide	Asnaashari, Dastmalchi, and Javadzadeh (2018); Khan et al. (2012)
*Alhagi camelorum*	Leguminosae	Camelthorn	Aerial parts	Quercetin, kaempferol, isorhamnetin, β‐sitosterol, lupeol, phenolic acids, lignans, and alkaloids.	Shaker, Mahmoud, and Mnaa (2010)
*Allium sativum*	Amaryllidaceae	**Toum or garlic**	Bulb	Flavonoids, Tannins, Terpenoids, and gallic acid	Doukanı et al. (2021)
*Aloe ferox*	Aloaceae	Bitter aloe or Cape aloe	Leaves	Anthraquinones (aloin and aloe‐emodin), polysaccharides (acemannan), flavonoids, and saponins	Bentahar et al. (2016)
*Aloe vera* Miller	Xanthorrhoeaceae	Aloe	Aerial part (leaves)	Hydroxybenzoic acid, benzoic acid, catechin, gallic acid, chlorogenic acid, coumaric acid, epicatechin, rosmarinic acid, syringic acid, trans‐ferrulic acid; quercitin, cinamic acid, hesperidin, and sinapic acid	Fedoul et al. (2022)
*Althaea officinalis*	Malvaceae	Marshmallow	Roots, flowers, and leaves	Mucilage, quercetin, kaempferol, rutin, caffeic acid, and chlorogenic acid	Šutovská et al. (2009)
*Artemisia herba‐alba*	Asteraceae	White wormwood	Aerial parts	Essential oil; tricyclene, artemisia triene, α‐thujene, α‐pinene, camphene, sabinene, β‐pinene, cis‐pinane, myrcene, α‐phellandrene, α‐terpinene, ortho‐cymene, limonene, 1,8‐cineole, E‐ β‐ocimene, γ‐terpinene, artemisia ketone, α‐pinene oxide, sabinene trans hydrate, α‐thujone, β‐thujone, trans‐pinan‐2‐ol, chrysanthenone, terpinol, camphor, β‐pinene oxide, trans β‐dihydro terpineol, trans β‐terpineol, terpinen‐4‐ol, thuj‐3‐en‐10‐al, p‐cymen‐8‐ol, α‐terpineol, trans piperitol, p‐cymen‐9‐ol, trans chrysanthenyl acetate, piperitone, cis chrysanthenyl acetate, neo‐3‐ thujyl acetate, α‐terpinen‐7‐al, γ‐terpinen‐7‐al, α‐terpinyl acetate, β‐elemene, E‐caryophyllene, 9‐epi E–caryophyllene, and ô‐muurolene	Houti et al. (2023)
*Berberis vulgaris*	Berberidaceae	Zereshk	Aerial parts	Flavonoids, alkaloids, tannins, and saponins	
*Brassica oleracea var. botrytis*	Brassicaceae	Cauliflower	Aerial parts	Glucosinolates, vitamin C, vitamin K, quercetin, kaempferol, and sulforaphane	Kononenko, Mirzaliev, and Ostapets (2018); Sudharameshwari and Ayshwarya (2017)
*Brassica oleracea* var. capitata	Brassicaceae	Cabbage	Leaves	Gallic acid, chlorogenic acid, catechin hydrate, vanillic acid, (−) epicatechin, *p‐*coumaric acid, rutin hydrate, and rosmarinic acid	Rahman et al. (2022)
*Carlina acanthifolia*	Asteraceae	Baranbar	Roots	Sesquiterpene lactones, flavonoids, and triterpenes,	Ivancheva, Nikolova, and Tsvetkova (2006)
*Centaurea chamaerhaponticum*	Asteraceae	Pontic cornflower or Pontic knapweed	Aerial parts	Tannins, sesquiterpene lactones, and flavonoids	Bakhtaoui, Lakmichi, Chait, and Gadhi (2014)
*Centaurium erythraea*	Gentianaceae	Centaury	Aerial parts	Secoiridoid glycosides (sweroside and gentiopicroside), apigenin, luteolin, quercetin, mangiferin, and isomangiferin.	Bentahar et al. (2016); Đorđević et al. (2017)
*Ceratonia siliqua*	Fabaceae	Carob tree	Pods, seeds, and leaves	Gallic acid, condensed tannins, fiber, and proteins	Benmansour et al. (2020); Elaoufi et al. (2022)
*Cicorium intybus*	Asteraceae	Chicory	Roots and leaves	Sesquiterpene lactones, flavonoids, phenolic acids, chicoric acid, inulin, coumarins, inulin, and caffeic acid	Nouir et al. (2023); Saxena et al. (2011); Saxena, Sulakhiya, and Rathore (2014)
*Cinnamomum verum*	Lauraceae	Cinnamon	Bark	Essential oil; camphene, β‐pinene, sabinene, myrcene, 1,4‐cineole, limonene, cis‐β‐ocimene, trans‐β‐ocimene, p‐cymene, linalool, γ‐terpinene, α‐terpineol, piperitone, geraniol, (E)‐cinnamaldehyde, (Z)‐cinnamaldehyde, eugenol, (E)‐cinnamyl acetate, eugenyl acetate, and benzyl benzoate	Narayanankutty et al. (2021)
*Globularia alypum*	Globulariaceae	Globe daisy	Leaves, roots, and flowers	Alkaloids, flavonoids, and phenolic acids, iridoids, secoiridoids, and phenylethanoid glycosides	Nouir et al. (2023); Taghzouti et al. (2016)
*Glycyrrhiza glabra*	Leguminosae	Licorice	Rhizomes	Tannins, flavonoids, reducing compounds, saponins, coumarins, triterpenes, and sterols	Benbott et al. (2021)
*Curcuma longa*	Zingiberaceae	Turmeric	Rhizomes	Starch, glucosides, mucilage, and terpenoids	Benmeziane‐Derradji and Aoun (2022)
*Helianthemum kahiricum*	Cistaceae	Egyptian sunrose or Egyptian rockrose	Aerial parts	Kaempferol‐3‐D‐(6‐O‐trans‐p‐coumaroyl) glucopyranoside and kaempferol‐3‐O‐(3,6 ‐di‐O‐trans‐p‐coumaroyl) glucopyranoside	Ayoola et al. (2016); Mouffouk et al. (2023)
*Helianthemum lippii*	Cistaceae	Sunrose rockrose	Aerial parts	Flavonoids, phenolic acids, tannins, terpenoids, and alkaloids	Alsabri et al. (2014, 2013)
*Juniperus communis*	Cupressaceae	Common “juniper” or simply “juniper.”	Aerial parts	Alpha‐pinene, beta‐pinene, limonene, myrcene, quercetin, kaempferol, rutin, tannins, essential oils (monoterpenes and sesquiterpenes), and pectin	Bais et al. (2014); Pramanik et al. (2007)
*Juniperus phoenicea*	Cupressaceae	Red juniper	Leaves	Alpha‐pinene, beta‐pinene, limonene, and myrcene, flavonoids, tannins, and essential oils	Ali et al. (2015); Hoogerwerf and Pasricha (2006)
*Lagenaria siceraria*	Cucurbitaceae	Bottle gourd or calabash	Aerial parts	Coniferyl alcohol, ferulic acid, p‐coumaric acid, flavonoids, saponins, and tannins	Manchala (2019); Mehboob et al. (2022)
*Lavandula stoechas*	Lamiaceae	Spanish lavender or French lavender	Leaves	Camphor and fenchone	Ben Mansour et al. (2022)
*Lawsonia inermis*	Lythraceae	Henna	Leaves	2‐hydroxy‐1,4‐naphthoquinone and suavissimoside R1	Muheyuddeen et al. (2023)
*Mangifera indica*	Anacardiaceae	Mango	Leaves	Stigmasterol and β‐sitosterol, and pentagalloyl glucose	Kagbo and Aduku (2015); Mahmoud et al. (2022)
*Matricaria chamomilla*	Asteraceae	Chamomile	Flowers	Alkaloids, total tannins, condensed tannins, gallic tannins, mucilage, flavonoids, saponins, and glycosides	Online et al. (2018)
*Mentha microphylla*	Lamiaceae	Corsican mint or tiny‐leafed mint	Leaves	1,2‐dihydroxy‐p‐menth‐4(8)‐en‐3‐one, 4‐hydroxy‐p‐mentha‐1,8‐dien‐3‐one, 4‐methoxy‐p‐mentha‐1,8‐dien‐3‐one, 3‐acetoxy‐6‐hydroxy‐p‐mentha‐1,8‐diene.	Mahmoud (2005)
*Mentha spicata*	Lamiaceae	Spearmint	Leaves	Caffeic, chlorogenic, rosmarinic, vanillic, syringic, *p*‐coumaric, ferulic, rosmarinic, 3,4‐dihydroxybenzoic, ferulic acid, and diosmetin, diosmin, diosmin‐7‐glucoside, thymonin, 5,6,4′‐Trihydroxy‐7,3′‐dimethoxyflavone, Eriocitrin, luteolin glucoside, isorhoifolin, luteolin, apigenin, quercetin, luteolin, scopoletin, catechin, epicatechin, rutin, myricetin, luteolin, apigenin, and naringenin	Fatiha et al. (2015)
*Myrtus communis*	Myrtaceae	Common myrtle or simply myrtle	Leaves	α‐Pinene, Limonene, 1,8‐cineol, Linalool oxide, Linalool, Fenchyl alcohol, α‐Terpineol, cis‐pinocarveol, Nerol, Mertenyl acetate, Geraniol, Linalyl acetate, P‐menth‐1‐enol, trans—Pinocarveyl acetete, α‐Terpinyl acetate, Neryl acetate, Methyl eugenol, Trans caryophyllene, α‐Humulene, α‐Pinene, Limonene, 1,8‐cineol, Linalool oxide, Linalool, Fenchyl alcohol, α‐Terpineol, cis‐pinocarveol, Nerol, Mertenyl acetate, Geraniol, Linalyl acetate, P‐menth‐1‐enol, trans—Pinocarveyl acetete, α‐Terpinyl acetate, Neryl acetate, Methyl eugenol, Trans caryophyllene, α‐Humulene, α‐Pinene, Limonene, 1,8‐cineol, Linalool oxide, Linalool, Fenchyl alcohol, α‐Terpineol, cis‐pinocarveol, Nerol, Mertenyl acetate, Geraniol, Linalyl acetate, P‐menth‐1‐enol, trans—Pinocarveyl acetete, α‐Terpinyl acetate, Neryl acetate, Methyl eugenol, Trans caryophyllene, α‐Humulene, α‐Pinene, limonene1,8‐cineol, linalool oxide, linalool, fenchyl alcohol, α‐terpineolcis‐pinocarveol, nerol, mertenyl acetate, geraniol, linalyl acetate, p‐menth‐1‐enol, trans—pinocarveyl aceteteα‐terpinyl acetate, neryl acetate, methyl eugenol, trans caryophyllene, and α‐humulene	Nassar et al. (2010)
*Nigella sativa*	Ranunculaceae	Black seed	Seeds	Volatile oil: *p*‐Cymene, α‐thujene, sabinene, thymoquinone, carvacrol, trans‐sabinene hydrate, γ‐terpinene, longifolene and α‐longipinene	Benkaci‐Ali, Baaliouamer, and Meklati (2005)
*Olea europaea*	Oleaceae	Olive	Leaves and fruit	Oleuropein, hydroxytyrosol, oleanolic acid, maslinic acid, and quercetin	Mahdavi et al. (2020); Musa et al. (2021)
*Oreganum majorana*	Lamiaceae	Sweet marjoram or simply marjoram	Aerial parts	Rosmarinic acid, apigenin, luteolin, caffeic acid, carvacrol, thymol, saponins, and essential oils	Dorman and Deans (2000); Mathias (1994)
*Oreganum syriacum*	Lamiaceae	Syrian oregano	Leaves	Rosmarinic acid, flavonoids, phenolic acids, carvacrol, thymol, essential oils, and saponins	El‐Meligy et al. (2017); Mesmar et al. (2022)
*Origanum compactum Benth*	Lamiaceae	Oroccan oregano or compact oregano	Leaves	Flavonoids, phenolic acids, carvacrol and thymol, saponins, and essential oils	Bakhtaoui, Lakmichi, Chait, and Gadhi (2014); Hayani et al. (2022)
*Opuntia ficus‐indica*	Cactaceae	Prickly pear or cactus pear	Fruit, pads (Cladodes), and seeds	Betacyanins, betaxanthins, dietary fiber, flavonoids, phenolic acids, and tannins	Trachtenberg and Mayer (1981)
*Osyris quadripartite*	Santalaceae	African sandalwood	Leaves, roots, stems, and bark,	Flavonoids, tannins, and saponins	Kale, Bambare, and Bhale (2023)
*Phoenix dactylifera*	Arecaceae	Date palm	Pulp and palm sap	Sugar, dietary fibers, sodium, potassium, magnesium, zinc, and iron	Abdellaziz et al. (2014)
*Pistacia lentiscus*	Anacardiaceae	Mastic tree	Aerial parts	Arachidonic acid, oleanolic acid, ursolic acid, masticadienonic acid, isomasticadienolic acid, and β‐sitosterol	Boutemine et al. (2018); Calder (2001)
*Plantago major*	Plantaginaceae	Broad leaf Plantain or Plantain	Leaf	Flavonoids, alkaloids, terpenoids, phenolic acid derivatives, iridoid glycosides, fatty acids, polysaccharides, and vitamins	Adom et al. (2017)
*Portulaca oleracea*	Portulacaceae	Purslane	Aerial parts	Kaempferol, apigenin, luteolin, myricetin, and quercetin, polysaccharides, and α‐linolenic acid	Karimi, Hosseinzadeh, and Ettehad (2004); Tijani, Temitayo, and Farombi (2022)
*Punica granatum*	Punicaceae	Pomegranate	Peel	Quinic acid, gallic acid, protocatechuic acid, catechin (+), caffeic acid, syringic acid, quercetin, kaempferol, and cirsiliol	Alimi et al. (2023)
*Rhus tripartite*	Anacardiaceae	Three‐leaved sumac or skunkbush sumac	Roots, stem, and leaves	Tannins, flavonoids, terpenoids, citric acid malic acid, and alkaloids	Benmansour et al. (2020); Shahat et al. (2016)
*Solanum nigrum*	Solanaceae	Black nightshade	Leaves, berries, roots, and stems	Saponins, flavonoids, alkaloids, phenolic acids, tannins, and steroids	Jainu and Devi (2004a); Zaghlool et al. (2019)
*Syzygium aromaticum*	Myrtaceae	Clove	Fleurs	Eugenol, gallic acid, β‐caryophyllene, vanillin, crategolic acid (maslinic acid), campesterol, kaempferol, rhamnetin, oleanolic acid, eugenitin, bicornin, eugenin, quercetin, and ellagic acid	Batiha et al. (2020)
*Trigonella foenum‐graecum*	Fabaceae	Fenugreek	Seed	Diosgenin, saponins, quercetin, apigenin, luteolin, galactomannans, and trigonelline	Jainu and Devi (2004b); Selmi et al. (2022)
*Curcuma longa*	Zingiberaceae	Turmeric	Rhizomes	Starch, glucosides, mucilage, and terpenoids	(Benmeziane–Derradji & Aoun, 2022)
*Vitis vinifera*	Vitaceae	Grapevine	Fruit, leaves, and seed	Quercetin, kaempferol, catechins, proanthocyanidins, resveratrol, tartaric acid, malic acid, citric acid, carotenoids, anthocyanins, proanthocyanidins, phenolic acids, and tannins.	Felicio, Santos, and Gonçalez (2001); Saadaoui et al. (2020)
*Zingiber Officinale*	Zingiberacea	Ginger	Rhizomes	Carbohydrates, volatile oils, sterols, triterpenoids, alkaloids, kaempferol, quercetin‐hexoside, quercetin‐3‐O‐rutinoside, syringic acid, (+)‐Catechin, ferulic acid, ellagic acid, quercetin, salicylic acid, gallic acid, quercetin‐hexoside, and kaempferol‐3‐O‐rutinoside	Saiah et al. (2018); Zaghlool et al. (2015)
*Ziziphus lotus*	Rhamnaceae	Lotus jujube or lote tree	Fruits, leaves, seeds, and bark	Tannins, betulinic acid, oleanolic acid, quercetin, kaempferol, saponins, arabinoxylans, and arabinogalactans	Bakhtaoui, Lakmichi, Megraud et al. (2014b); Wahida, Abderrahman, and Nabil (2007)

## 4 Results

### 4.1 Medicinal Plants for Peptic Ulcer Disease

#### 4.1.1 Abelmoschus esculentus

*A. esculentus*, commonly known as okra or lady's finger. *A. esculentus* has been traditionally used in certain herbal medicine systems for gastrointestinal health (Ortaç et al. 2018). *A. esculentus* contains mucilage, a gel‐like substance that is known for its demulcent properties. Mucilage can form a protective layer over the gastric mucosa, helping to soothe irritation and inflammation in the stomach lining (Naim et al. 2015). Additionally, several studies illustrated Okra is rich in antioxidants, including flavonoids, polyphenols, and vitamin C, which can help neutralize harmful free radicals in the body (Joshi et al. 2011). Oxidative stress is known to play a role in the development and progression of gastric ulcers, and the antioxidant compounds in okra may help protect the gastric mucosa from oxidative damage (Ortaç et al. 2018).

#### 4.1.2 *Acacia senegal* L.

*A. senegal* (gum Arabic) was traditionally used to treat many disorders, including stomach illness. Researchers linked the constituents of gum Arabic and its efficacy in maintaining the normal status of gastric mucosa (Khedr 2017). The high percentage of polysaccharides in the aqueous solution of gum Arabic may explain its gastroprotective potential against gastritis and be a promising therapy in treating gastric cancer. Previous studies showed that oral gavage of 500 mg gum Arabic/kg to Wistar rats has reduced the ulcer index by 15% in the acute gastric ulcer model (Salama and Mariod 2018). Recent studies showed that oral administration of gum Arabic at doses of 500 mg/kg and 1000 mg/kg exhibits anti‐ulcer activity in rats ulcerated by ethanol. Scientists explained the efficacy of gum Arabic against ethanol gastritis in terms of the cytoprotective potency of gum Arabic to gastric cells as well as its free radical scavenging capacity (Salama and Mariod 2018).

#### 4.1.3 Aegle marmelos

*A. marmelos*, commonly known as bael, is a tree from the Rutaceae family widely used in traditional ayurvedic medicine for gastrointestinal problems. Different parts of *A. marmelos* like the fruit, leaves, and roots have exhibited anti‐ulcer effects in animal models against gastric ulcers induced by ethanol and NSAIDs. Proposed gastroprotective mechanisms of bael include antisecretory activity to reduce gastric acid secretion and antioxidant properties from phenolic compounds that help prevent oxidative damage to gastric tissue (Venthodika et al. 2021). Specific phytochemicals isolated from bael like the coumarin marmin, obtained from leaves, can inhibit the growth of *H. pylori* and help protect gastric epithelial cells. Another key coumarin named Angeline, abundant in bael leaves, has shown anti‐secretory, cytoprotective, and antioxidant effects in the gastric mucosa in rats. However, despite the promising gastroprotective results in preliminary preclinical studies, rigorous randomized controlled trials in humans evaluating safety and efficacy are lacking for bael preparations. Further research warrants exploring its anti‐ulcer effects in clinically relevant models along with bioactivity‐guided fractionation to identify its active constituents (Zhang et al. 2020).

#### 4.1.4 Aerva persica

*A. persica*, commonly known as desert cotton, has been used traditionally in various cultures for its medicinal properties diuretic, and demulcent properties (Chawla et al. 2013). Several biological activities have been reported from *A. persica* such as antioxidant, antibacterial, antifungal, hypoglycemic, and anti‐ulcer effects (Mehboob et al. 2022). Based on research into the anti‐ulcer effects of *A. persica* roots, it was found that administration of ethanol extract from these roots reduced ethanol‐induced hemorrhagic necrosis in the stomach of rats, as observed in histopathological evaluations of the gastric mucosa (Vasudeva et al. 2012).

#### 4.1.5 Alhagi camelorum

*A. camelorum* belongs to the Leguminosae family and is used in folk medicine to treat some gastric diseases (Zarei, Ashtiyani, and Vaezi 2014). The whole plant has historically been used to treat a variety of ailments, including rheumatic, metabolic, GI, and liver issues, diuretics, migraines, fever, warts, and rash (Shaker, Mahmoud, and Mnaa 2010). There have been reports of this plant's anti‐ulcerogenic, gastroprotective, ureteral stone ejection, antidiarrheal, antinociceptive, and antioxidant properties. Previous research has demonstrated that an aqueous extract of *A. camelorum* greatly protected and inhibited secretory processes in rat stomach mucosa (Gharibn and Mard 2007). Additionally, rat stomach mucosa experimental research on *A. maurorum* extract showed strong mucosal protection and antisecretory activity (Shaker, Mahmoud, and Mnaa 2010).

#### 4.1.6 Allium sativum

Garlic (Amaryllidaceae) has been used as a digestive stimulant and ulcer remedy in traditional medicine (Shang et al. 2019). Safavi, Shams‐Ardakani, and Foroumadi (2015) highlight its potential benefits, particularly in protecting against NSAID‐induced ulcers in rats. Aged garlic extract was found to safeguard against these ulcers by preventing the depletion of the important antioxidant glutathione and reducing lipid peroxidation. Moreover, it exhibited inhibitory effects on the growth of *H. pylori* bacteria and their adhesion to gastric epithelial cells in laboratory settings. The active compounds responsible for these gastroprotective effects were identified as organosulfur compounds, namely S‐allylcysteine and S‐allylmercaptocysteine, found in garlic. Despite promising preclinical findings, the efficacy of garlic in treating peptic ulcer disease (PUD) patients lacks substantial clinical validation. Thus, further clinical studies are warranted to elucidate the therapeutic potential of garlic in managing PUD (Shang et al. 2019).

#### 4.1.7 Aloe ferox

*A. ferox* (Aloaceae), also known as bitter aloe or Cape aloe, has been traditionally used for various medicinal purposes, including potential therapeutic effects on gastric ulcers (Eamlamnam et al. 2006). *A. ferox* contains compounds such as anthraquinones, flavonoids, and polysaccharides, which have been shown to possess anti‐inflammatory properties. Chronic inflammation in the stomach lining can exacerbate gastric ulcers *and A. ferox* may help mitigate this inflammatory response (Bentahar et al. 2016). Additionally, the gel‐like sap (latex) obtained from the leaves of *A. ferox* has demulcent properties, meaning it forms a protective coating over the gastric mucosa. This protective barrier may help soothe irritation and inflammation in the stomach lining and promote the healing of gastric ulcers. Furthermore, some studies suggest that *A. ferox* may stimulate the production of gastric mucin, a glycoprotein that forms a protective layer over the stomach lining. By enhancing mucin production, *Aloe ferox* may help strengthen the gastric mucosal barrier and reduce the risk of ulcer formation (Borra, Lagisetty, and Mallela 2011).

#### 4.1.8 *Aloe vera* Miller

The leaf pulp of the succulent *A. vera* plant (Xanthorrhoeaceae) has a long history of medicinal use for gastrointestinal problems in traditional medicine. In animal models, *A. vera* gel has exhibited gastroprotective effects against gastric ulcers through proposed mechanisms like antioxidant activity, increasing gastric mucus secretion, and stimulating the production of prostaglandins and bicarbonate (Martínez‐Burgos et al. 2022). Bioactive compounds identified in *A. vera* gel including polysaccharides like acemannan and anthraquinones such as aloin, can stimulate mucus cell proliferation and exert antioxidant effects to protect the gastric mucosa. Small clinical trials in humans have also revealed some beneficial effects of *A. vera* syrup in reducing abdominal pain, gastric acidity, and ulcer symptoms in peptic ulcer patients. However, larger‐scale randomized placebo‐controlled trials are required to conclusively validate the efficacy and safety of *Aloe vera* preparations as an adjunct or alternative therapy for peptic ulcers and gastritis (Ogidi and Enenebeaku 2023).

#### 4.1.9 Althaea officinalis

*A. officinalis*, commonly known as marshmallow, has been used traditionally in herbal medicine for various gastrointestinal issues, including gastric ulcers (Ghavi 2015). The plant's roots, flowers, and leaves have been employed to treat a variety of illnesses including peptic ulcers, gastritis, ventricular ulcer, sore throat, asthma, bronchitis, and respiratory diarrhea. According to previous reports in the literature, aqueous extracts and polysaccharides in this plant present medicinal attributes (Šutovská et al. 2009). Furthermore, Marshmallow root contains high levels of mucilage, a gel‐like substance that forms a protective layer over the stomach lining. This mucilage may help soothe and protect the gastric mucosa, potentially reducing irritation and promoting ulcer healing. Additionally, Deters et al. have demonstrated that aqueous extracts and polysaccharides from *A. officinalis* roots can effectively stimulate epithelial cells, supporting the plant's traditional use as a tissue‐regenerating remedy for inflamed mucous membranes (Asnaashari, Dastmalchi, and Javadzadeh 2018; Deters et al. 2010).

#### 4.1.10 Artemisia herba‐alba

*A. herba‐alba*, commonly referred to as white wormwood or desert wormwood, holds potential as a treatment for gastric ulcers. This plant, known for its medicinal properties, has been utilized in various cultures over time. Multiple studies have explored its ability to manage gastric ulcers, uncovering intriguing results (Gacem et al. 2020). Evidence suggests that *A. herba‐alba* possesses anti‐ulcerogenic properties, meaning it can help prevent the formation of ulcers in the stomach lining. Its effects are thought to stem from a blend of actions, including anti‐inflammatory, antioxidant, and cytoprotective mechanisms. Research has indicated that extracts derived from *A. herba‐alba* can mitigate the development of gastric ulcers induced by factors such as alcohol, nonsteroidal anti‐inflammatory drugs (NSAIDs), and stress in animal models. Moreover, its anti‐inflammatory attributes may aid in reducing the inflammation associated with gastric ulcers, thus facilitating the healing process (Abushwereb 2016). Additionally, the antioxidant compounds present in *A. herba‐alba* may shield the gastric mucosa from oxidative damage, a common factor implicated in the onset and progression of gastric ulcers (Abdel Jaleel, Abdallah, and Gomaa 2016).

Despite encouraging preliminary findings, further investigation, particularly clinical trials involving human subjects, is necessary to comprehensively assess the effectiveness and safety of *A. herba‐alba* in treating gastric ulcers. Moreover, delving into the specific bioactive compounds responsible for their anti‐ulcer effects and determining optimal dosage regimens would offer valuable insights into its therapeutic capabilities.

#### 4.1.11 Berberis vulgaris

*B. vulgaris* (Berberidaceae) has been used in Ayurveda, Iranian, and Chinese medicine for treating gastrointestinal disorders (Shahdadian et al. 2023). Berberine, an alkaloid from *Berberis vulgaris*, exhibited anti‐ulcerogenic properties through inhibition of gastric acid secretion, stimulation of mucin secretion, and antioxidant effects in rats. Berberine also prevented NSAID‐induced intestinal injury by inhibiting inflammation and oxidative stress. Berberis's potential as an adjuvant to standard PUD therapy needs more investigation (Imenshahidi and Hosseinzadeh 2019).

#### 4.1.12 *Brassica oleracea* var. botrytis

*B. oleracea* var. botrytis, commonly known as cauliflower, has shown therapeutic effects on gastric ulcers (Kononenko, Mirzaliev, and Ostapets 2018). Research indicates that extracts from *B. oleracea*, including var. botrytis, have gastroprotective properties against peptic ulcers (Kononenko, Mirzaliev, and Ostapets 2018). Studies have demonstrated that the aqueous fraction of *B. oleracea* botrytis offers high protection against gastric ulcers, with approximately 95% efficacy (Atta, Nasr, and Mouneir 2005; Kononenko, Mirzaliev, and Ostapets 2018). Additionally, the lipophilic extracts of *B. oleracea* have shown significant anti‐ulcerogenic activity, promoting the healing of gastric ulcers in animal models (Sudharameshwari and Ayshwarya 2017). These findings suggest that *B. oleracea*, along with other varieties of *B. oleracea*, could be beneficial in the management and treatment of gastric ulcers due to their gastroprotective and ulcer‐healing properties.

#### 4.1.13 *Brassica oleracea* var. capitata

Both green and red cabbage varieties of *B. oleracea* have traditionally been used as part of folk remedies for healing stomach ulcers and gastrointestinal problems (Ray et al. 2021). Fresh cabbage juice has exhibited gastroprotective effects against ethanol, NSAID, stress, and cysteamine‐induced gastric ulcers and lesions in rat models through antioxidant mechanisms. Cabbage contains antioxidant flavonoids like kaempferol that help preserve the gastric mucosal barrier by stimulating mucus secretion and preventing oxidative damage to gastric tissue (Harsha et al. 2017; Hemmami et al. 2023). Another key flavonoid from cabbage, anthocyanin, has also demonstrated anti‐ulcer properties including antioxidant free radical scavenging, antisecretory effects on gastric acid, and cytoprotective activity in animal models. Crude extracts, juices, and purified bioactive compounds from cabbage thus seem to promote ulcer healing and gastritis treatment through combined antioxidant, antisecretory, mucoprotective, and anti‐inflammatory mechanisms. Further studies isolating and characterizing the specific phytochemicals responsible for cabbage's gastroprotective effects are warranted to understand its pharmaceutical potential for peptic ulcer disease (Kononenko et al. 2023).

#### 4.1.14 Carlina acanthifolia

*C. acanthifolia* is a perennial plant that is common in Eastern Serbia's hills and mountains and belongs to the Asteraceae family (Leporatti and Ivancheva 2003). *C. acanthifolia* root is used for its diuretic, anti‐inflammatory therapy for the urinary tract, antioxidant, antimicrobial, and anti‐ulcer agent (Ivancheva, Nikolova, and Tsvetkova 2006). Traditionally have been used in herbal medicine for various gastrointestinal issues due to their potential anti‐inflammatory and gastroprotective properties. Previous studies on the efficacy of the root's essential oil showed a noteworthy dose‐dependent gastroprotective effect in rats that had completed a stress gastric ulcer test caused by ethanol (Đorđević et al. 2007).

#### 4.1.15 *Centaurea chamaerhaponticum* Bal.

*C. chamaerhaponticum*, commonly known as Pontic cornflower or Pontic knapweed, is a species of flowering plant in the family Asteraceae (Kaunitz 1999). *C. chamaerhaponticum* belongs to the Centaurea genus, which includes several species known for their medicinal properties including ulcer gastric diseases. Some species within the Centaurea genus have been studied for their anti‐inflammatory, antioxidant, and gastroprotective effects, which could potentially have implications for the treatment or prevention of gastric ulcers. Previous studies' results showed that crude extracts of *C. chamaerhaponticum* possess significant gastroprotective and antisecretory properties which prevent gastric ulceration induced by different necrotizing agents (Bakhtaoui, Lakmichi, Chait, and Gadhi 2014).

#### 4.1.16 Centaurium erythraea

*C. erythraea*, commonly known as common centaury, has been traditionally used in herbal medicine for various purposes including gastric ulcers (El Menyiy et al. 2021). Common centaury contains bitter principles, including iridoids such as gentiopicrin and amarogentin, which stimulate bitter receptors in the mouth and digestive tract. Bitter substances have been shown to enhance digestion by stimulating the secretion of saliva, gastric juices, and bile, which can aid in the breakdown and absorption of nutrients (Đorđević et al. 2017). Further, some studies have suggested that herbal remedies containing bitter principles may have gastroprotective effects, including promoting the secretion of protective mucus in the stomach lining and enhancing the integrity of the gastric mucosa (Bentahar et al. 2016). While direct evidence for *C. erythraea* gastroprotective effects is lacking, its bitter properties may contribute to similar mechanisms of action.

#### 4.1.17 *Ceratonia siliqua* L.

*C. siliqua*, commonly known as carob tree or St. John's bread, is a species of flowering evergreen tree in the Fabaceae family, that exhibits therapeutic effects on gastric ulcers (Bakhtaoui, Lakmichi, Chait, and Gadhi 2014). Research has shown that the plant contains various beneficial compounds such as polyphenols, flavonoids, and phenolic acids, which contribute to its antioxidant, anti‐inflammatory, and anti‐ulcer properties (Elaoufi et al. 2022). Studies have demonstrated that the carob pods' extracts can inhibit hyaluronidase activity, reduce oxidative damage, and protect against gastric mucosal damage induced by HCl/ethanol (Rtibi et al. 2016). Additionally, *C. siliqua* extracts have been found to limit colonic damage in rats with ulcerative colitis, showcasing anti‐inflammatory and antioxidant effects (Lakkab et al. 2018). The plant's bioactive components also exhibit antibacterial, antifungal, and antidiabetic activities, suggesting its potential as a natural alternative for various health conditions, including gastrointestinal issues like gastric ulcers (Benmansour et al. 2020).

#### 4.1.18 Cicorium intybus

*C. intybus*, commonly known as chicory exhibits therapeutic effects on gastric ulcers due to its gastroprotective properties. The plant's root extract demonstrates a gastroprotective effect by reducing gastric secretions and enhancing the defense barrier of the gastric mucosa in previous studies (Krylova et al. 2015). Various pharmacological studies confirm the plant's therapeutic value, showcasing activities such as anti‐ulcer, antioxidant, and free radical scavenging properties (Saxena, Sulakhiya, and Rathore 2014). Additionally, the hydroalcoholic extract of *C. intybus* roots show significant anti‐ulcer and antioxidant activities, reducing gastric juice volume and acidity while increasing pH levels, indicating its potential in ulcer treatment (Saxena et al. 2011). The plant's rich phytoconstituents, including inulin, flavonoids, sesquiterpene lactones, and vitamins, contribute to its diverse medicinal benefits (Das, Vasudeva, and Sharma 2016). Additionally, Chicory is rich in antioxidants, including vitamins A and C, flavonoids, and phenolic compounds. These antioxidants help neutralize free radicals and oxidative stress, which can damage the gastric mucosa and increase the risk of ulcer formation (Krylova et al. 2015).

#### 4.1.19 Cinnamomum verum

*C. verum*, also known as true cinnamon, belongs to the Lauraceae family and is esteemed for its multifaceted medicinal properties. Across traditional healing practices, *C. verum* has been integral for centuries, notably in regions like South Asia and the Middle East, revered for its diverse therapeutic applications. The bark and essential oil of *C. verum* have been traditionally employed to address an array of health concerns, including gastrointestinal disorders such as indigestion, bloating, and diarrhea. These remedies often utilize decoctions or infusions derived from *C. verum* bark, renowned for its soothing and gastroprotective effects (Ranasinghe et al. 2013). Numerous scientific inquiries have illuminated the therapeutic potential of *C. verum*. Essential oils extracted from *C. verum* have demonstrated potent antimicrobial, antifungal, and antioxidant properties, suggesting their efficacy in combating various infectious and inflammatory conditions (Rao and Gan 2014). Additionally, research has underscored the anti‐ulcerative properties of *C. verum* extracts, particularly aqueous solutions, showcasing their potential to mitigate gastric ulceration in experimental models (Bandara, Uluwaduge, and Jansz 2012). Furthermore, *C. verum* has exhibited promising capabilities in modulating inflammatory pathways, exerting antisecretory effects, and bolstering the body's antioxidative defenses, indicative of its potential as a therapeutic agent for a spectrum of ailments (Sharma et al. 2022).

#### 4.1.20 *Globularia alypum* L.

*G. alypum*, a member of the Globulariaceae family, is a common plant in the Mediterranean. *G. alypum* has long been utilized in traditional medicine to treat various disorders caused by oxidative stress (Ziyyat et al. 1997). The research on *G. alypum* highlights its gastroprotective efficacy along with various other beneficial properties. Studies have shown that extracts of *G. alypum* possess significant antioxidant activities, which can help protect against oxidative damage induced by radicals like AAPH (Ouffai et al. 2022). Additionally, the plant exhibits anti‐inflammatory properties, which can contribute to gastroprotection (Nouir et al. 2023). Furthermore, the phytochemical composition of *G. alypum* includes bioactive compounds like verbascoside, known for their potential gastroprotective effects (Hajji et al. 2021). Moreover, numerous studies have shown that *G. alypum* leaf and flower extracts are rich in secondary metabolites such as polyphenols and iridoids (Amessis‐Ouchemoukh et al. 2014; Taghzouti et al. 2016). Those compounds are well known for their antioxidant properties, anti‐ulceration, anti‐inflammation (Boutemak, Safta, and Ayachi 2015), and anticancer activities (Es‐Safi et al. 2006). These findings suggest that *G. alypum* has the potential to offer gastroprotective benefits, making it a promising candidate for further exploration in gastroprotection research. Nevertheless, further research, including preclinical and clinical studies, is needed to elucidate its efficacy and safety for the prevention and treatment of peptic ulcers and other gastrointestinal disorders.

#### 4.1.21 Glycyrrhiza glabra

Licorice derived from the roots of *G. glabra* (Leguminosae) has a long history of use in traditional medicine systems as an expectorant and treatment for peptic ulcers and other gastrointestinal problems. Several deglycyrrhizinated licorice preparations containing glycyrrhizin, isoliquiritigenin, and polysaccharides have exhibited gastroprotective effects in animal models and human studies of peptic ulcer disease (Maqbool et al. 2023). Proposed mechanisms for licorice's anti‐ulcer properties include increasing the production of gastric mucus and prostaglandins, antioxidant effects, and inhibiting *H. pylori* colonization. The triterpene glycyrrhizin can bind to intracellular steroidal receptors leading to increased prostaglandin formation and cytoprotective effects in the gastric mucosa. Licorice flavonoids such as liquiritigenin can also stimulate mucin release from gastric epithelial cells through prostaglandin E2 pathways in vitro. Further research needs to definitively characterize the active anti‐ulcer constituents in licorice and elucidate their molecular mechanisms of gastroprotection. Nonetheless, the clinical studies combined with preclinical data suggest that licorice possesses therapeutic potential for peptic ulcer treatment warranting more rigorous randomized controlled trials to validate its efficacy and safety in humans (El‐Saber Batiha et al. 2020; Maqbool et al. 2023; Patel and Khetani 2021).

#### 4.1.22 *Helianthemum kahiricum* L.

*H. kahiricum* is a species of flowering plant belonging to the family Cistaceae. *H. kahiricum* has shown therapeutic effects on gastric ulcers (Alsabri et al. 2014). Studies have highlighted its anti‐ulcer properties, including antioxidant effects and protection against ethanol‐induced ulcers (Mouffouk et al. 2023). Additionally, the plant exhibited significant anti‐inflammatory and anti‐microbial activities, contributing to its overall therapeutic potential (Ayoola et al. 2016; Olaitan Balogun et al. 2022). Chemical investigations have identified compounds in *H. kahiricum* that play a role in its medicinal properties, such as kaempferol derivatives with antimicrobial activity. Furthermore, the plant's extracts have demonstrated gastric ulcer healing effects, modulation of cytokines, and tissue regeneration, showcasing its potential in treating chronic gastric ulcers (Ayoola et al. 2016). Overall, *H. kahiricum* shows promise as a natural remedy for gastric ulcers due to its diverse pharmacological properties and therapeutic effects.

#### 4.1.23 *Helianthemum lippii* L.

*H. lippii* commonly known as sunrose rockrose or white‐flowered cistus, is a flowering plant species belonging to the family Cistaceae (Ibtissam and Djahra 2022). *H. lippii* has been extensively studied for its therapeutic effects, including its anti‐ulcer properties. Research has shown that *H. lippii* methanolic extracts exhibit significant anti‐ulcer activity, with doses of 250 mg/kg and 500 mg/kg demonstrating substantial inhibition of gastric lesions in rats induced with ethanol, comparable to the standard anti‐ulcer drug ranitidine (Alsabri et al. 2013). The protective effect of *H. lippii* against ethanol‐induced gastric mucosal lesions is attributed to its increase in antioxidant activity, highlighting its potential in treating gastric ulcers (Alsabri et al. 2014). Additionally, the phytochemical screening of *H. lippii* revealed the presence of compounds like flavonoids and tannins, which contribute to its therapeutic properties, including anti‐ulcer effects (Alsabri et al. 2013). These findings position *H. lippii* as a promising natural remedy for gastric ulcers.

#### 4.1.24 Juniperus communis

*J. communis* has been extensively studied for its therapeutic effects, including its anti‐ulcer properties. Research has shown that the leaf extract of *J. communis* significantly inhibits various types of gastric ulcerations in rats and promotes healing of ulcers, comparable to the standard drug ranitidine (Pramanik et al. 2007). Additionally, *J. communis* contains essential oils, phenolic compounds, and various chemical constituents that contribute to its pharmacological effects, such as anti‐inflammatory and analgesic properties (Al‐Snafi 2018). Traditionally used for its medicinal benefits, *J. communis* is known for its anti‐inflammatory, antiseptic, and astringent properties, making it potentially effective in treating abdominal disorders (Bais et al. 2014). Furthermore, the plant's extract and essential oils exhibit antibacterial, antioxidant, and anti‐inflammatory activities, highlighting its potential in treating various diseases, including ulcers (Fierascu et al. 2018).

#### 4.1.25 *Juniperus phoenicea* L.

*J. phoenicea* also known as red juniper. In folk medicine, this plant is considered a remedy that is commonly used in many countries for the treatment of diarrhea, bronchitis, rheumatism, eczema, hemorrhoids, dysmenorrheal, and ulcer gastric (Mansour et al. 2023). The essential oil of *J. phoenicea* seeds has been identified as a potent source of natural antioxidants and antimicrobial agents, and ulcer gastric diseases. Consistent with this, different authors reported that essential oils have high curative characteristics and fewer side effects than chemical drugs, acting through the modulation of oxidative stress and the targeting of inflammatory parameters. Several studies illustrated, that the oral administration of the essential oil of *J. phoenicea* has potent anti‐ulcer activity, which justifies the ethnomedical claims about its significant gastroprotective effect, as evaluated by the significant antioxidant activity because it reduces the MDA level and increases the GSH, GST, GPx, CAT, and SOD levels (Ali et al. 2015; Hoogerwerf and Pasricha 2006). This effect may be related to an increase in gastric mucosal defense mechanisms. As a result, the effect of the essential oil and its low toxicity requires further study to elucidate the mechanism of action and isolate the active principle.

#### 4.1.26 *Lagenaria siceraria* L.

*L. siceraria*, commonly known as bottle gourd or calabash, is a vine plant belonging to the gourd family (Cucurbitaceae) (Ahmed, Nedi, and Yimer 2022). The aqueous extracts of *L. siceraria* have shown therapeutic effects on gastric ulcers. Studies have demonstrated the anti‐gastric ulcer activity of *L. siceraria*, with significant reductions in gastric acidity, increased pH levels, and enhanced gastric wall mucus production, indicating gastroprotective potential (Saeed et al. 2022). Additionally, *L. siceraria* is known for its antioxidant properties due to compounds like flavonoids, saponins, and tannins, which contribute to its anti‐ulcer action (Mehboob et al. 2022). The anti‐ulcer efficacy of a methanolic extract of *L. siceraria* fruits was examined in Wistar rats using pylorus ligation, asprin, cold‐restraint stress, and ethanol ulcer models. MELS reduced stomach volume, free acidity, ulcer index, and total acidity significantly, indicating that *L. siceraria* fruit extract may have anti‐ulcer action. As a result of histological assessment investigations, it was shown that *L. siceraria* is both safe and effective in the treatment of stomach ulcers. Additionally, Bottle gourd is rich in dietary fiber, particularly soluble fiber. Soluble fiber can help regulate digestion, promote bowel regularity, and improve overall gastrointestinal health. Adequate fiber intake is associated with a reduced risk of certain gastrointestinal conditions, including gastric ulcers according to several studies (Manchala 2019).

#### 4.1.27 Lavandula stoechas

*L. stoechas*, commonly known as Spanish lavender or French lavender, is a species of flowering plant in the Lavandula genus (Şahinler et al. 2022). *L. stoechas* has a long history of traditional medicinal use for various gastrointestinal issues, including stomachaches (Elrherabi et al. 2023). The aqueous extract demonstrated anti‐ulcer activity in ethanol‐induced gastric and duodenal ulcers in experimental animals, comparable to standard drugs like omeprazole and ranitidine. Additionally, the extract exhibited antimicrobial properties against *Proteus Mirabilis*, indicating its potential in combating ulcer‐related infections. Some studies suggest that lavender essential oil may have cytoprotective effects on the gastric mucosa. These effects involve enhancing the secretion of gastric mucus, increasing mucosal blood flow, and stimulating the production of prostaglandins, which help protect the stomach lining from harmful factors (Javed et al. 2020). Additionally, several studies illustrated the effects of anxiolytic and stress‐reducing *L. stoechas* chronic stress and anxiety are known risk factors for the development of peptic ulcers. Lavender has been traditionally used for its calming and stress‐relieving properties. By reducing stress and anxiety levels, lavender may indirectly help prevent ulcers and promote gastrointestinal health (Javed et al. 2020).

#### 4.1.28 *Lawsonia inermis* L.

*L. inermis*, commonly known as henna, exhibits therapeutic effects on gastric ulcers. Studies have shown that *L. inermis* possesses anti‐ulcer properties (Elansary et al. 2020; Moutawalli et al. 2023). The plant's leaves, when administered in a nano formulation, demonstrated the ability to prevent ulcer formation in rats induced with aspirin, showcasing significant improvements in various parameters and biochemical activities (Muheyuddeen et al. 2023). Additionally, *L. inermis* contains active compounds like flavonoids, phenols, alkaloids, and tannins, which contribute to its pharmacological effects, including anti‐inflammatory, analgesic, and wound‐healing properties (Muheyuddeen et al. 2023). The plant's extracts have been found to have antioxidant activity and high flavonoid content, further supporting its potential therapeutic benefits for gastric ulcers. This collective evidence underscores the promising role of *L. inermis* in the management of gastric ulcers (Mohammed et al. 2022).

#### 4.1.29 Mangifera indica

*M. indica* (Anacardiaceae), commonly known as mango, is being used in folk medicine for the treatment of various diseases including gastric ulcers (Sahu, Martha, and Pradhan 2017). Several studies, it was demonstrated that the ethanolic and aqueous extract of leaves of *M. indica* exhibited remarkable gastroprotective activity in pylorus ligation, aspirin plus pylorus ligation, and ethanol‐induced gastric ulcer models in experimental Wistar rats. In addition, Kagbo and Aduku (2015) illustrated that *M. indica* may have an equal extent of therapeutic effect gastroprotective effect with the largely used well‐known anti‐gastric ulcer drug Ranitidine. Moreover, other studies suggested glucosylxanthone from *M. indica* is a gastroprotective agent responsible for the anti‐ulcer effect (Carvalho et al. 2007).

#### 4.1.30 Matricaria chamomilla

The dried flowers of *M. chamomilla* (Asteraceae) have been traditionally used as a digestive aid and treatment for gastritis and peptic ulcers (Srivastava and Gupta 2015). Apigenin, a bioactive flavone isolated from chamomile, has been shown to dose‐dependently reduce gastric injury and inflammation induced by *H. pylori* infection in vitro. It exhibits these gastroprotective effects by suppressing pro‐inflammatory cytokines like TNF‐α and IL‐8 while also inhibiting the activity of *H. pylori* cytotoxic virulence factors (El Joumaa and Borjac 2022). Beyond anti‐inflammatory effects, extracts of *M. chamomilla* flowers demonstrate direct antibacterial activity against *H. pylori* growth in vitro (Cvetanović et al. 2015). Animal studies have also revealed chamomile oil can accelerate the healing of acetic acid‐induced chronic gastric lesions in rats through antisecretory mechanisms and antioxidant effects. Specific constituents of chamomile flowers like the terpenoids bisabolol and chamazulene, and flavonoids like apigenin, quercetin, and patuletin have been postulated to mediate its gastroprotective properties, but require further investigation. Overall, chamomile possesses the broad therapeutic potential for the protection and treatment of peptic ulcers through its combination of antibacterial, anti‐inflammatory, antisecretory, mucoprotective, and antioxidant effects (Singh et al. 2023).

#### 4.1.31 Mentha microphylla

*M. microphylla*, commonly known as Corsican mint or tiny‐leafed mint, is a low‐growing perennial herbaceous plant belonging to the Lamiaceae family (Brahmi et al. 2017). *M. microphylla* was originally used as a medicinal herb to treat stomachache, and it is commonly used in the form of tea as a home remedy to stimulate digestion, alleviate stomach pain, and treat biliary disorders, dyspepsia, enteritis, flatulence, gastritis, and gastric acidities. Some studies suggest that mint extracts can enhance the production of mucus in the stomach lining, which serves as a protective barrier against stomach acid and irritants. This mucosal protection may help prevent or alleviate gastrointestinal disorders. Moreover, several studies illustrated the aqueous fraction of *M. microphylla* showed a prominent protective effect (100% protection) in Wistar rats, which suggests the beneficial use of infusion or decoction teas of Mentha as protective against peptic ulcers (Thompson Coon and Ernst 2002). Additionally, certain compounds found in mint, such as menthol and menthone, have been investigated for their potential to prevent and heal ulcers in the stomach and intestines (Atta, Nasr, and Mouneir 2005). These compounds may help by reducing gastric acid secretion and enhancing mucosal defense mechanisms.

#### 4.1.32 Mentha spicata

Studies have explored the potential of *M. spicata*, commonly known as spearmint, in treating peptic ulcer diseases, especially in Northern Africa where such ulcers are common and traditional remedies often include herbal treatments like spearmint (Anwar et al. 2019). Research indicates that *Mentha spicata* may offer benefits for individuals with peptic ulcers, as it is thought to have anti‐inflammatory and gastroprotective properties, potentially aiding in symptom relief and ulcer healing (Singh et al. 2023). Investigations in Northern Africa have shown promising results, demonstrating Spearmint's ability to reduce ulcer formation and alleviate symptoms in animal models. Furthermore, its historical use in traditional medicine in the region suggests its perceived effectiveness for gastrointestinal issues. However, further research, particularly well‐designed clinical trials involving human participants, is necessary to confirm the efficacy of *M. spicata* in managing peptic ulcer diseases in Northern Africa. Additionally, understanding its mechanisms of action and determining optimal dosages would enhance its therapeutic application in this context (Anwar et al. 2019).

#### 4.1.33 *Myrtus communis* L.

*M. communis*, belonging to the Myrtaceae family, is commonly used in traditional medicine as a decoction to treat stomach and gastrointestinal disorders such as diarrhea, constipation, and peptic ulcer (Flamini et al. 2004). Moreover, distinct studies have previously demonstrated the disinfectant, antiseptic, antimicrobial, and antioxidant capacities of *M. communis* essential oils, as well as their potential to fight several diseases. In addition, a previous study highlighted the anti‐ulcer capacity of orally administered methanolic extracts of *M. communis* leaves (Alipour, Dashti, and Hosseinzadeh 2014), in experimental models of gastric ulceration (Ben Mansour et al. 2022). Further, several studies have demonstrated the positive effect of plant essential oils through the modulation of inflammatory mediators, antisecretory, and antioxidative stress defense. More importantly, the treatment of animals with MMEO successfully inhibited oxidative damage and reversed the impairment of the antioxidant system in the intestinal mucosa according to previous studies (Mansour et al. 2022).

#### 4.1.34 Nigella sativa

*N. sativa* (black seed) is an annual herbaceous plant that belongs to the Ranunculaceae family. It grows up to 30–60 cm tall with finely divided leaves and small black seeds. The seeds of *N. sativa* have been utilized traditionally in North Africa for the treatment of various gastrointestinal disorders, including stomach ulcers. The therapeutic effects of *N. sativa* have been attributed to its various bioactive compounds, such as thymoquinone, thymohydroquinone, and thymol (Alam et al. 2023). Thymoquinone, a major active component of *N. sativa*, has been demonstrated to exhibit anti‐ulcer activity in several experimental models of gastric ulceration. In a study by Bukar et al. (2017), thymoquinone was found to protect against ethanol‐induced gastric mucosal injury in rats. Similarly, Zeren et al. (2016) reported that thymoquinone inhibited gastric acid secretion and increased mucus production in rats with indomethacin‐induced gastric ulcers. In addition, thymoquinone has been shown to exhibit antimicrobial activity against *H. pylori*, a bacterium commonly associated with stomach ulcers (Tabassum and Ahmad 2021). Other bioactive compounds in *N. sativa*, such as thymohydroquinone and thymol, have also been reported to possess anti‐ulcer activity. Thymohydroquinone has been shown to reduce gastric acid secretion and increase mucus production in rats with ethanol‐induced gastric ulcers (Paseban et al. 2020; Zakir et al. 2022). Moreover, thymol has been demonstrated to exhibit anti‐inflammatory and antioxidant activity, which may contribute to its gastroprotective effects (Liu et al. 2022). In vitro studies have also confirmed the anti‐ulcer activity of *N. sativa* extracts. For instance, Geetha and Anitha (2022) reported that an ethanolic extract of *N. sativa* inhibited the growth of *H. pylori* in vitro. Similarly, Bukar et al. (2017) found that an aqueous extract of *N. sativa* protected against ulceration in rats with acetic acid‐induced gastric lesions.

#### 4.1.35 Olea europaea

*O. europaea* (Olive), commonly known as the olive tree, has been traditionally used for various medicinal purposes, including the treatment of gastric ulcers (Unissa et al. 2022). *O. europaea* exhibits therapeutic effects in treating gastric ulcers by demonstrating gastroprotective, anti‐ulcer, antioxidant, and anti‐inflammatory properties. Studies have shown that olive leaf extract significantly reduces ulcer index, protects the gastric mucosa, and regulates inflammatory cytokines, such as IL‐1β, IL‐2, IL‐4, IL‐6, IL‐10, and TNF‐α (Musa et al. 2021). Additionally, olive extract decreases histological changes in the small intestine, enhances gastrointestinal function, and reduces intestinal permeability, as indicated by decreased plasma D‐lactate concentration. Furthermore, olive oil extracted from *Opuntia ficus‐*indica seeds has been found to efficiently protect the gastric mucosa, stimulate mucus production, and accelerate the healing process of ethanol‐induced ulcers (Mahdavi et al. 2020; Musa et al. 2021).

#### 4.1.36 Oreganum majorana

*O. majorana*, commonly known as sweet marjoram or simply marjoram, is an aromatic herb belonging to the mint family (Lamiaceae) (Al‐Howiriny et al. 2009). The aerial parts of marjoram or the oil are used for “strengthening of the stomach,” and to cure acute and chronic gastritis by containing various chemical constituents including phenolic glycosides, flavonoids, phenolic compounds, tannins, and essential oil (Dorman and Deans 2000; Mathias 1994). Several studies have demonstrated that pretreatment with marjoram leads to a significant improvement in the depleted levels of NP‐SH concentration, indicating the strong involvement of endogenous sulfhydryl compounds (SHs) in the gastroprotective effects of marjoram extract. NP‐SH compounds are known for their role in scavenging oxygen‐derived free radicals and regulating the production and quality of mucus. Chemical constituents found in marjoram, such as tannins, flavonoids, and volatile oils, are believed to prevent the loss of gastric mucus and NP‐SH. These compounds have been shown to eliminate free radicals generated as a result of ethanol‐induced mucosal ulceration. Tannins, flavonoids, and volatile oils have been associated with antioxidant properties in previous research (Allen et al. 1984; Andreo et al. 2006).

#### 4.1.37 *Oreganum syriacum* L

*O. syriacum*, commonly known as Syrian oregano, is a herbaceous plant belonging to the mint family (Lamiaceae) (El‐Meligy et al. 2017). *O. syriacum* has demonstrated therapeutic effects on gastric ulcer. Several studies showed that oral administration of the ethanolic extract of *O. syriacum* significantly reduced gastric damage in experimental rats (Mesmar et al. 2022). In another study investigating the anti‐ulcerogenic activity of various plant extracts in prophylactic and curative models, the oral administration of *O. syriacum* ethanolic extract to rats with absolute‐ethanol‐induced gastric damage was shown to have a similar effect to the anti‐ulcer drug lansoprazole in the prophylactic model only (El‐Meligy et al. 2017). The ethanolic extract also exhibited high free‐radical scavenging activity, further supporting the antioxidant potential of *O. syriacum* and its protective and healing role in ethanol‐induced gastric ulceration. Furthermore, the extract from *O. syriacum* has been shown to protect the gastric mucosa from ethanol‐induced injury by inducing cyclooxygenase‐2 (COX‐2) expression and decreasing oxidative stress in the stomach.

#### 4.1.38 Origanum compactum

*O. compactum* (Lamiaceae), commonly known as Moroccan oregano or compact oregano, is a species of oregano native to North Africa, particularly Morocco (Bouyahya et al. 2020). This Moroccan medicinal plant, also known as Zaatar, has been traditionally used to treat various ailments, including digestive issues (Bouyahya et al. 2016). Studies have highlighted the plant's antimicrobial, antioxidant, and anti‐inflammatory properties, which are crucial in addressing gastric ulcer pathophysiology (Hayani et al. 2022). The essential oils extracted from *O. compactum* have demonstrated significant antibacterial activity against various strains, including *E. coli* and *S. aureus*, indicating its potential in combating infections related to gastric ulcers (Bakhtaoui, Lakmichi, Chait, and Gadhi 2014). Additionally, the plant's bioactive compounds, such as thymol and carvacrol, contribute to its pharmacological mechanisms, supporting its efficacy in treating gastric ulcers and other related conditions. Further research on the therapeutic potential of *O. compactum* in gastric ulcer management is encouraged to explore its full range of benefits (Bakhtaoui, Lakmichi, Chait, and Gadhi 2014).

#### 4.1.39 O. ficus‐indica

*O. ficus‐indica*, commonly known as prickly pear or cactus pear. The cladodes of *O. ficus indica* are used in folk medicine in the treatment of gastric mucosal diseases (Galati et al. 2001; Saag et al. 1975). Several researchers demonstrated in experimental Wistar rats, that the prophylactic therapy with lyophilized cladodes of *O. ficus indica* inhibits the ulcerative effect of ethanol. One could assume that the mucilages of *O. ficus indica* are responsible for the effects that have been noted. The mucilage could stop the necrotizing agent from penetrating the stomach mucosa. Maybe it creates a shield and prevents the deep necrotic lesions and the large‐scale ethanol‐induced surface epithelium exfoliation (Trachtenberg and Mayer 1981). It is likely that the mucilage, a high molecular weight acid polysaccharide primarily composed of arabinogalactan and galacturonic acid (Saag et al. 1975), can work in concert with the stomach mucosa's defense mechanisms.

#### 4.1.40 Osyris quadripartita

*O. quadripartita*, commonly known as African sandalwood, is a species of flowering plant in the family Santalaceae (Abebaw, Mishra, and Gelayee 2017). *O. quadripartita* (OQ) has been scientifically validated for its therapeutic effect on gastric ulcers. Studies have shown that OQ possesses significant anti‐ulcer activity (Babu et al. 2020). The plant extract demonstrated a dose‐dependent and time‐dependent reduction in gastric ulcer index in both pylorus ligation‐induced and ethanol‐induced ulcer models, comparable to standard drugs like ranitidine and sucralfate. Additionally, OQ's oral median lethal dose (LD_50_) was estimated to be higher than 2000 mg/kg, indicating its safety profile (Kale, Bambare, and Bhale 2023). Furthermore, OQ's anti‐ulcer properties have been attributed to the presence of secondary metabolites like flavonoids, tannins, and saponins in the plant extract (Jainu and Devi 2004b). These findings validate the traditional use of OQ in Ethiopian folk medicine for treating peptic ulcers and highlight its potential as a natural remedy for gastric ulcer management.

#### 4.1.41 *Phoenix dactylifera* L

*P. dactylifera*, commonly known as the date palm, is a member of the Arecaceae family and is renowned for its versatile medicinal properties. In traditional medicine, *P. dactylifera* has been utilized for centuries, particularly in the Middle East and North Africa, for its gastroprotective efficacy. Date palm pulp and pulp palm sap have been traditionally used as decoctions to alleviate various stomach and gastrointestinal disorders, including diarrhea, constipation, and peptic ulcers (Stielow et al. 2023).

Several studies have highlighted the therapeutic potential of *P. dactylifera*. Essential oils derived from *P. dactylifera* have demonstrated notable disinfectant, antiseptic, antimicrobial, and antioxidant properties, suggesting their potential efficacy in combating a range of diseases (Al‐Zoreky and Al‐Taher 2019). Additionally, research has indicated the anti‐ulcerative effects of orally administered aqueous extract of *P. dactylifera*, particularly in experimental models of gastric ulceration (Abdullahi and Chinedu 2022). Furthermore, *P. dactylifera* has shown promising results in modulating inflammatory mediators, exhibiting antisecretory effects, and enhancing the body's antioxidative stress defense mechanisms (Al‐Shahib and Marshall 2003).

#### 4.1.42 *Pistacia lentiscus* L.

*P. lentiscus* (Anacardiaceae) is a flowering plant growing in the Mediterranean area. It is traditionally used in the treatment of gastrointestinal upsets and gastric ulcers (Al‐Habbal, Al‐Habbal, and Huwez 1984; Duru et al. 2003). The anti‐ulcer and anti‐inflammatory activities of PLFO could be due to its phytochemical compounds such as fatty acid, tocopherols, sterols, phenolic components, and terpenoids. The major fatty acids in PLFO can inhibit the activity of COX‐1 and COX‐2 (Ben Khedir et al. 2016). These enzymes are involved in the production of inflammatory mediators such as arachidonic acid‐derived eicosanoids (such as prostaglandins, thromboxanes, and leukotrienes) (Calder 2001). At sufficient levels, long‐chain n‐3 polyunsaturated fatty acids (PUFAs), such as α‐linoleic acid found in PLFO, inhibit arachidonic acid metabolism. PUFAs can also reduce the levels of pro‐inflammatory cytokines (such as IL‐6 and TNF‐α) and the expression of adhesion molecules implicated in the interactions between leukocytes and endothelial cells during the infiltration process. Moreover, Boutemine et al. (2018) highlighted its protective effect. As pre‐treatment, PLFO could be prescribed to patients predisposed to have a gastric ulcer such as people who consume a lot of alcohol and anti‐inflammatory drugs orally.

#### 4.1.43 Plantago major

*P. major*, commonly referred to as greater plantain, has garnered attention for its potential to treat various gastrointestinal ailments, including peptic ulcer diseases. Particularly in regions like North Africa, where traditional herbal remedies hold significance, greater plantain has been historically utilized for its medicinal properties (Hemmami et al. 2023). Research indicates that *P. major* may possess anti‐inflammatory and mucoprotective properties, which could be advantageous in relieving symptoms and facilitating healing in individuals with peptic ulcers. Studies have demonstrated promising findings, suggesting that extracts derived from *P. major* can mitigate ulcer formation and shield the gastric mucosa from damage caused by substances like alcohol and NSAIDs (Ragheb, Ibrahem, and Shalaby 2021). Moreover, its wide availability and minimal toxicity enhance its appeal as an herbal treatment option. Nonetheless, further investigation, particularly through clinical trials involving human participants, is imperative to comprehensively grasp the effectiveness and optimal use of *P. major* in managing peptic ulcer diseases, not only in North Africa but also in other regions (Bahadeen Aref et al. 2023).

#### 4.1.44 Portulaca oleracea

*P. oleracea* (Portulaceae, common name purslane). Kaempferol, apigenin, luteolin, myricetin, and quercetin are major flavonoids in *P. oleracea* that prevent gastrointestinal diseases (Farkhondeh and Samarghandian 2019). Studies have shown that *P. oleracea* extract and juice can effectively reduce ulcerative colitis (UC) symptoms by decreasing disease activity index (DAI) scores and inflammatory cell infiltration (Tijani, Temitayo, and Farombi 2022). Additionally, *P. oleracea* juice has been found to inhibit pyroptosis by reducing the expression of NLRP3 inflammasome, repairing intestinal barrier dysfunction, and upregulating tight junction proteins. Furthermore, the anti‐ulcer properties of *P. oleracea* have been linked to its ability to enhance the mucosa‐bicarbonate barrier, reduce gastric ulceration, and positively impact antioxidant defense systems (Karimi, Hosseinzadeh, and Ettehad 2004). Additionally, purslane contains omega‐3 fatty acids, flavonoids, and polysaccharides, which have demonstrated anti‐inflammatory properties. Chronic inflammation is a contributing factor to the development and progression of gastric ulcers. By reducing inflammation in the stomach lining, purslane may aid in the healing process of gastric ulcers (Kumar et al. 2010).

#### 4.1.45 Punica granatum

Studies have explored the potential of *P. granatum*, or pomegranate, in treating and preventing stomach ulcers due to its traditional medicinal use and perceived health benefits. It is believed that *P. granatum* exhibits gastroprotective properties through various mechanisms, including its antioxidant, anti‐inflammatory, and mucoprotective actions (Muhialdin et al. 2023). Research indicates that extracts from this fruit can reduce the development of stomach ulcers induced by alcohol, NSAIDs, and stress in animal models. The high antioxidant content, particularly polyphenols like ellagic acid and punicalagin, may help protect the stomach lining from oxidative damage, a factor often linked to ulcer formation. Despite promising initial results, further investigations, including human clinical trials, are needed to fully comprehend the effectiveness of *P. granatum* in treating stomach ulcers. Additionally, identifying specific bioactive compounds and determining optimal dosage regimens would enhance its therapeutic application (Alimi et al. 2023).

#### 4.1.46 Rhus tripartita

*R. tripartita*, also known as three‐leaved sumac or skunkbush sumac, is a shrub native to North Africa and parts of the Middle East. It is primarily known for its use in traditional medicine, particularly in North African traditional healing systems, specifically for ulcer gastric diseases (Shahat et al. 2016). Research indicates that *R. tripartita* may possess anti‐inflammatory, antioxidant, and cytoprotective effects properties, which could be advantageous in relieving symptoms and facilitating healing in individuals with peptic ulcers. Studies have demonstrated promising findings, suggesting that extracts derived from *R. tripartita* major can mitigate ulcer formation and shield the gastric mucosa from damage caused by substances like alcohol and NSAIDs (Ben Mansour et al. 2022). Nevertheless, further research, including clinical trials, is needed to validate the efficacy and safety of *R. tripartita* and related species for medicinal use in treating or preventing gastrointestinal disorders.

#### 4.1.47 Solanum nigrum

*S. nigrum*, commonly known as black nightshade, is a plant that has been traditionally used in various cultures for its medicinal properties (Jainu and Devi 2006). Certain components of black nightshade, such as alkaloids and saponins, may possess gastroprotective properties (Zaghlool et al. 2019). These compounds may help strengthen the gastric mucosal barrier, inhibit gastric acid secretion, and promote tissue regeneration, thereby protecting against gastric ulcers. Previous reports indicated that *S. nigrum* fruits possess beneficial activity as an anti‐ulcer, and antioxidant‐promoting agent in rats (Jainu and Devi 2004a; Kumar et al. 2001). It has been reported earlier that aerial parts of *S. nigrum* are believed to offer their anti‐ulcer action through acid and peptic suppression in aspirin‐induced ulcerogenesis in rats (Akhtar and Munir 1989). The preliminary phytochemical screening of SNE revealed the presence of tannins, alkaloids, carbohydrates, saponins, volatile oils, and anthocyanins. Previous studies proved that anthocyanins possess significant antioxidant, anti‐ulcer, and ulcer healing activity in experimental ulcer models. Additionally, saponins, tannins, and volatile oils of some plants are also known to possess anti‐ulcer activity (Gohar and Zaki 2014).

#### 4.1.48 Syzygium aromaticum

*S. aromaticum*, commonly known as clove, belongs to the Myrtaceae family and is esteemed for its diverse medicinal properties. Across traditional healing practices, *S. aromaticum* has been integral for centuries, particularly in regions like Southeast Asia and the Indian subcontinent, revered for its versatile therapeutic applications. The aromatic buds and essential oil of *S. aromaticum* have been traditionally employed to address an array of health concerns, including dental issues, digestive disorders, and respiratory ailments. These remedies often utilize clove infusions or decoctions derived from the buds, renowned for their soothing and antimicrobial effects (Batiha et al. 2020; Maggini et al. 2024). Numerous scientific inquiries have illuminated the therapeutic potential of *S. aromaticum*. Essential oils extracted from *S. aromaticum* have demonstrated potent antibacterial, antifungal, and antioxidant properties, suggesting their efficacy in combating various infectious and inflammatory conditions (Liñán‐Atero et al. 2024; Pandey et al. 2024). Additionally, research has underscored the anti‐ulcerogenic activity of polyphenol‐rich extract of clove buds, showcasing their potential in alleviating gastrointestinal disorders and ulcers (Issac et al. 2015). Furthermore, *S. aromaticum* has exhibited promising capabilities in modulating inflammatory pathways, antisecretory effects, and bolstering the body's antioxidative defenses, indicative of its potential as a therapeutic agent for a spectrum of ailments (Barboza et al. 2018; Okasha 2007).

#### 4.1.49 Trigonella foenum‐graecum

*Trigonella foenum‐graecum*, commonly known as fenugreek, exhibits therapeutic effects on gastric ulcers (Jainu and Devi 2004b). Studies have shown that the aqueous and ethanolic extracts of *Trigonella foenum‐graecum* seeds significantly protect against ethanol‐induced gastric ulcers in rats, demonstrating gastroprotective properties comparable to omeprazole (Selmi et al. 2022). These extracts have been found to improve the condition of the gastric mucosa, enhance antioxidant enzyme activities like SOD and CAT, and reduce oxidative stress markers such as MDA and H_2_O_2_, thereby aiding in the prevention of gastric damage induced by harmful agents like ethanol (Afroz, Rahman, and Kamal 2017). Additionally, some studies have suggested that fenugreek may have mucoprotective effects on the gastric mucosa, helping to enhance the protective barrier and reduce the risk of ulcer formation. These effects could be attributed to the presence of polysaccharides and other bioactive compounds in fenugreek seeds (Bhat, Sharma, and Singh 2018).

#### 4.1.50 Curcuma longa

Turmeric, derived from the rhizome of *C. longa* belonging to the Zingiberaceae family, has a long history of use in both Ayurvedic and Chinese medicinal practices for the treatment of gastric ulcers (Akaberi, Sahebkar, and Emami 2021). Within turmeric, the active compound curcumin has garnered attention for its remarkable gastroprotective properties. Studies have demonstrated its effectiveness against various ulcer‐inducing agents such as indomethacin, ethanol, stress, and pylorus ligation in rat models. Curcumin exerts its protective effects through multiple mechanisms including antioxidant activity, inhibition of gastric acid secretion, mucosal protection, and anti‐*H. pylori* activity (against *H. pylori*) (Mohammadi et al. 2022).

Additionally, curcumin has been found to work synergistically with conventional treatments such as omeprazole and probiotics like *Lactobacillus acidophilus* to enhance its anti‐ulcer effects. Despite its potential benefits, curcumin's poor bioavailability poses a challenge to its therapeutic application. Ongoing research focuses on developing innovative drug delivery systems to improve curcumin's bioavailability, thereby maximizing its efficacy as an anti‐ulcer agent (Beiranvand 2022).

#### 4.1.51 *Vitis vinifera* L

*V. vinifera* commonly known as grapevine, is widely cultivated for its fruit. *Vitis vinifera* L. (Vitaceae) is one of the largest fruit crops in the world is a natural medicinal plant used since antiquity in traditional folk medicine in many countries in the world, including countries of northern Africa (Saadaoui et al. 2020). The leaves of *V. vinifera* L have been utilized traditionally in North Africa for the treatment of various gastrointestinal disorders, including stomach ulcers. it has been demonstrated that gastroprotective mechanisms are based on the ability to rapidly strengthen the main defensive mediators against gastric lesions which also can play a pivotal role in regulating gastric secretion and motility (Zhao et al. 2015). These gastroprotective mechanisms participate in gastric defense by inhibiting gastric acid secretion, stimulating the release of mucus and bicarbonate, and increasing blood flow on gastric mucosal (Matsuda et al. 2002; Wallace and Miller 2000). In a study by Saadaoui et al. (2020), acute pre‐treatment of rats with the grapevine leaves extracts or with omeprazole induced a significant reduction in the volume and acidity of gastric juice as well as the ulcer index. This gastroprotection seems to be related to, at least in part, antioxidant potential and also anti‐secretory acid effects. In addition, the presence of high rates of bioactive compounds certainly contributes to the installation of these activities such as organic acids, carotenoids, lipids, enzymes, vitamins, terpenes, reducing or non‐reducing sugars, resveratrol, other stilbene derivatives, phenolics acids, flavonoids including flavonols, anthocyanins, and proanthocyanidins (Bombardelli and Morazzoni 1995; Felicio, Santos, and Gonçalez 2001; Şendoğdu et al. 2006).

#### 4.1.52 Zingiber Officinale

In North Africa, *Z. officinale*, commonly known as ginger, has garnered attention for its potential to treat peptic ulcer diseases, which are prevalent gastrointestinal ailments in the region. Traditional medicinal practices often incorporate herbs like ginger due to its perceived health benefits (Saiah et al. 2018). Studies suggest that ginger may possess anti‐inflammatory and gastroprotective properties, which could alleviate symptoms and aid in the healing of ulcers in the stomach and duodenum. Research conducted in North Africa has shown promising results, demonstrating ginger's ability to reduce ulcer formation and alleviate associated symptoms in animal models. However, further well‐designed clinical trials involving human participants are needed to confirm its efficacy in managing peptic ulcer diseases in the region. Additionally, exploring the mechanisms of action and determining optimal dosage regimens would provide valuable insights for its therapeutic use in this context (Bereksi et al. 2018; Zaghlool et al. 2015).

#### 4.1.53 *Ziziphus lotus* L.

*Z. lotus L*., also known as lotus jujube or lote tree, is a plant species belonging to the genus *Ziziphus*. Some related species within the Ziziphus genus have been studied for their potential gastroprotective effects (Bakhtaoui, Lakmichi, Megraud et al. 2014). It has many therapeutic actions, anti‐inflammatory activity by compounds found in Ziziphus species, such as flavonoids and triterpenoids, possess anti‐inflammatory properties. These compounds may help reduce inflammation in the gastrointestinal tract and protect the gastric mucosa from damage. Further, Ziziphus species contain antioxidants that help scavenge free radicals and reduce oxidative stress. By protecting against oxidative damage, these antioxidants may contribute to the maintenance of gastric health and the prevention of gastric ulcers. Research indicates that Ziziphus extracts may exhibit anti‐ulcer activity by reducing gastric acid secretion, inhibiting the activity of enzymes involved in ulcer formation, and promoting the healing of gastric ulcers. These effects contribute to the overall gastroprotective properties of Ziziphus species (Wahida, Abderrahman, and Nabil 2007).

## 5 Role of Plant‐Derived Bioactive Compounds in the Management of Peptic Ulcers

Peptic ulcers, encompassing both gastric and duodenal ulcers, manifest as erosions in the lining of the gastrointestinal tract and are commonly associated with factors like *H. pylori* infection, the use of nonsteroidal anti‐inflammatory drugs (NSAIDs), alcohol intake, smoking, and stress (Lanas and Chan 2017). Traditional treatment approaches for peptic ulcers mainly revolve around suppressing acid, eradicating H. pylori, and safeguarding the mucosa. However, these methods sometimes fall short, prompting a growing interest in complementary or alternative therapies, notably those involving plant‐derived bioactive compounds (Boakye‐Yiadom et al. 2021).

The role of plant‐derived bioactive compounds can be delineated into several key aspects.

### 5.1 Anti‐Inflammatory Properties

Plant‐derived bioactive compounds, including flavonoids, curcumin, and polyphenols, exhibit notable anti‐inflammatory properties. These compounds have been extensively studied for their ability to modulate the inflammatory response by targeting various pathways involved in inflammation. One of the primary mechanisms through which they exert their anti‐inflammatory effects is by inhibiting the synthesis of pro‐inflammatory mediators such as cytokines and prostaglandins (Prayoga et al. 2024).

Flavonoids, which are abundantly found in fruits, vegetables, and herbs, possess strong anti‐inflammatory activity. They interfere with the signaling pathways that lead to the production of inflammatory cytokines, such as tumor necrosis factor‐alpha (TNF‐α) and interleukins (IL), thereby attenuating the inflammatory response in the gastrointestinal mucosa. Additionally, flavonoids inhibit the activity of enzymes involved in the synthesis of prostaglandins, such as cyclooxygenase (COX), which further contributes to their anti‐ulcerogenic effects (Kononenko, Mirzaliev, and Ostapets 2018; Zhang et al. 2020).

Curcumin, a bioactive compound derived from turmeric, is renowned for its potent anti‐inflammatory properties. It exerts its effects by modulating multiple molecular targets involved in inflammation, including transcription factors, cytokines, and enzymes. Curcumin inhibits the activation of nuclear factor‐kappa B (NF‐κB), a key regulator of inflammation, and downregulates the expression of pro‐inflammatory cytokines like TNF‐α, IL‐1β, and IL‐6. Moreover, curcumin suppresses the activity of COX and lipoxygenase (LOX), enzymes responsible for the synthesis of prostaglandins and leukotrienes, respectively (Akaberi, Sahebkar, and Emami 2021; Mohammadi et al. 2022).

Polyphenols, another class of plant‐derived compounds found in foods such as green tea, berries, and nuts, possess potent anti‐inflammatory properties. They exert their effects by modulating various signaling pathways involved in inflammation, including the mitogen‐activated protein kinase (MAPK) pathway and the NF‐κB pathway. Polyphenols inhibit the activation of inflammatory transcription factors, leading to reduced expression of pro‐inflammatory genes. Additionally, they enhance the activity of endogenous antioxidant enzymes, thereby mitigating oxidative stress‐induced inflammation (Hemmami et al. 2023).

By targeting multiple pathways involved in inflammation, plant‐derived bioactive compounds effectively attenuate the inflammatory response within the gastrointestinal mucosa. This anti‐inflammatory activity plays a crucial role in facilitating the healing of peptic ulcers by reducing mucosal inflammation and promoting tissue repair. Moreover, by alleviating inflammation, these compounds also help alleviate symptoms associated with peptic ulcers, such as pain and discomfort, thereby improving the overall quality of life for affected individuals (Gündoğdu, Özbayer, and Kar 2023; Silvan et al. 2021).

### 5.2 Antioxidant Capacity

Oxidative stress is a key contributor to the pathogenesis of peptic ulcers, leading to mucosal damage and impaired healing. Reactive oxygen species (ROS) and free radicals generated during oxidative stress can cause oxidative damage to biomolecules such as lipids, proteins, and DNA, resulting in cellular dysfunction and tissue injury. In the gastrointestinal tract, oxidative stress disrupts the delicate balance between mucosal defense mechanisms and harmful factors, predisposing the mucosa to ulcer formation and delaying the healing process (Gündoğdu, Özbayer, and Kar 2023).

Plant‐derived antioxidants, including flavonoids, phenolic acids, and carotenoids, play a crucial role in combating oxidative stress and promoting ulcer recovery. These bioactive compounds possess the ability to neutralize free radicals and reactive oxygen species, thereby preventing oxidative damage to cellular components. Additionally, they reinforce cellular antioxidant mechanisms by enhancing the activity of endogenous antioxidant enzymes such as superoxide dismutase (SOD), catalase, and glutathione peroxidase (Silvan et al. 2021).

Flavonoids, abundant in various fruits, vegetables, and beverages such as tea and red wine, are potent antioxidants that scavenge free radicals and inhibit oxidative stress‐induced cellular damage. These compounds exert their antioxidant effects through multiple mechanisms, including direct radical scavenging, metal chelation, and modulation of antioxidant enzyme activity (Silvan et al. 2021; Zhang et al. 2020).

Phenolic acids, found in foods such as berries, nuts, and whole grains, also exhibit strong antioxidant properties. They effectively neutralize free radicals and inhibit lipid peroxidation, a process that contributes to mucosal damage and ulcer formation. Additionally, phenolic acids enhance the activity of endogenous antioxidant enzymes, further bolstering the cellular defense against oxidative stress (Alimi et al. 2023; Geetha and Anitha 2022).

Carotenoids, pigments present in colorful fruits and vegetables, act as potent antioxidants by scavenging free radicals and singlet oxygen species. These compounds help protect the gastric mucosa from oxidative harm and promote ulcer recovery by preventing lipid peroxidation and preserving cellular integrity (Pol et al. 2023).

By scavenging free radicals and bolstering cellular antioxidant defenses, plant‐derived antioxidants shield the gastric mucosa from oxidative damage and facilitate ulcer recovery. Their ability to counteract oxidative stress represents a promising therapeutic approach for the management of peptic ulcers, offering potential benefits in terms of both prevention and treatment. Incorporating antioxidant‐rich foods or supplements into the diet may help reduce oxidative stress and promote mucosal healing in individuals with peptic ulcers (Gündoğdu, Özbayer, and Kar 2023).

### 5.3 Cytoprotective Attributes

Certain mucilaginous compounds found in specific plants, notably licorice, and marshmallow, exhibit notable cytoprotective attributes. These compounds, known for their gel‐like consistency when exposed to water, play a crucial role in forming a protective barrier over the gastric mucosa (Maqbool et al. 2023; Zaghlool et al. 2015).

This protective barrier acts as a physical shield, effectively shielding the gastric mucosa from corrosive agents such as gastric acid and pepsin. By preventing direct contact between these aggressive factors and the delicate mucosal lining, mucilaginous compounds mitigate mucosal injury and reduce the risk of ulcer development (Zakir et al. 2022).

Licorice, for instance, contains glycyrrhizin and glycyrrhetinic acid, compounds known for their mucoprotective properties. These substances form a viscous coating over the gastric mucosa, providing a barrier against gastric acid and enhancing mucosal integrity. Similarly, marshmallows contain mucilage, a gel‐forming substance that adheres to the mucosal surface, forming a protective layer that helps prevent ulcer formation and promotes healing (Maqbool et al. 2023).

The cytoprotective effects of these mucilaginous compounds are particularly beneficial in conditions where mucosal integrity is compromised, such as in peptic ulcers. By bolstering the natural defense mechanisms of the gastric mucosa, these compounds contribute to the maintenance of mucosal health and facilitate ulcer healing. Overall, the cytoprotective attributes of mucilaginous compounds derived from plants like licorice and marshmallow represent a promising therapeutic approach for the management of peptic ulcers. By forming a protective barrier over the gastric mucosa, these compounds help prevent mucosal injury and promote ulcer healing, offering potential benefits in the prevention and treatment of peptic ulcer disease (Asnaashari, Dastmalchi, and Javadzadeh 2018).

### 5.4 Antimicrobial Potential

Several plant‐derived compounds demonstrate potent antimicrobial properties, specifically targeting *H. pylori*, a major contributor to peptic ulceration. Compounds like berberine and allicin have garnered attention for their effectiveness in inhibiting the growth of *H. pylori* and are considered potential adjunctive therapies in eradication protocols for this bacterium (Majumdar and Looi 2024).

Berberine, a naturally occurring alkaloid found in various medicinal plants such as goldenseal and barberry, exhibits significant antimicrobial activity against *H. pylori*. Berberine disrupts crucial microbial processes within *H. pylori* cells, including interference with cell wall synthesis and inhibition of essential enzymes, ultimately leading to growth inhibition and bactericidal effects (Imenshahidi and Hosseinzadeh 2019).

Allicin, a sulfur‐containing compound derived from garlic, is another potent antimicrobial agent effective against *H. pylori*. Allicin exerts its antimicrobial effects by disrupting multiple cellular processes in *H. pylori*, including inhibition of key enzymes involved in energy metabolism and cellular respiration. This disruption impairs bacterial growth and viability, making allicin a promising candidate for adjunctive therapy in *H. pylori* eradication regimens (Shang et al. 2019).

By targeting *H. pylori*, these plant‐derived compounds offer a complementary approach to conventional antibiotic therapy in the management of peptic ulcers associated with H. pylori infection. Incorporating these compounds into treatment protocols may enhance the efficacy of *H. pylori* eradication and reduce the risk of ulcer recurrence, thereby improving clinical outcomes for affected individuals (Harsha et al. 2017; Hemmami et al. 2023).

### 5.5 Facilitation of Gastric Mucosal Healing

Plant‐derived compounds such as epigallocatechin gallate (EGCG) play a pivotal role in facilitating the healing process of the gastric mucosa. EGCG, a polyphenol abundant in green tea, possesses remarkable properties that stimulate reparative mechanisms within the gastric mucosa, thereby expediting ulcer healing and reducing the likelihood of recurrent ulcers. One of the primary mechanisms through which EGCG facilitates gastric mucosal healing is by augmenting the proliferation of epithelial cells. Epithelial cells play a crucial role in maintaining the integrity of the gastric mucosa and are essential for the repair of damaged tissue. EGCG promotes the proliferation of these cells, accelerating the regeneration of the mucosal lining and facilitating the closure of ulcerative lesions (Ahmed, Nedi, and Yimer 2022; Zhang et al. 2020).

In addition to enhancing epithelial cell proliferation, EGCG also fosters angiogenesis, the process of new blood vessel formation. Angiogenesis is vital for supplying oxygen and nutrients to the site of injury, facilitating tissue repair and regeneration. By promoting angiogenesis, EGCG enhances the blood supply to the ulcerated area, thereby facilitating the delivery of essential nutrients and growth factors necessary for mucosal healing (Zhang et al. 2020).

Furthermore, EGCG possesses anti‐inflammatory and antioxidant properties, which contribute to its ability to promote gastric mucosal healing. By reducing inflammation and oxidative stress within the gastric mucosa, EGCG creates a favorable environment for tissue repair and regeneration, thereby expediting the healing process. Overall, plant‐derived compounds like EGCG play a crucial role in facilitating gastric mucosal healing by promoting epithelial cell proliferation, fostering angiogenesis, and mitigating inflammation and oxidative stress. By accelerating ulcer healing and restoring the structural integrity of the gastric mucosa, these compounds offer promising therapeutic benefits in the management of peptic ulcers, reducing the likelihood of ulcer recurrence and improving clinical outcomes for affected individuals (Rodríguez‐Negrete et al. 2024).

### 5.6 Acid Secretion Reduction

Certain plant‐derived compounds, such as deglycyrrhizinated licorice (DGL), play a crucial role in reducing gastric acid secretion, thereby alleviating symptoms associated with peptic ulcers and creating an environment conducive to mucosal healing. DGL, a form of licorice from which the compound glycyrrhizin has been removed, exerts its effects by modulating the activity of proton pumps in the stomach (Patel and Khetani 2021; Singh et al. 2023).

Proton pumps are responsible for the secretion of hydrochloric acid into the gastric lumen, contributing to the acidic environment necessary for digestion. By modulating the activity of these proton pumps, DGL effectively reduces the production of gastric acid, leading to decreased acidity within the stomach. The reduction in gastric acid secretion achieved by DGL offers several benefits in the management of peptic ulcers. Firstly, it helps alleviate symptoms such as abdominal pain, heartburn, and indigestion, which are often exacerbated by excessive gastric acid secretion. By lowering acid levels, DGL provides symptomatic relief, improving the overall quality of life for individuals with peptic ulcers. Moreover, the reduction in gastric acid secretion creates a more favorable environment for mucosal healing. Excessive gastric acid can exacerbate mucosal injury and delay the healing process by further damaging the already compromised gastric mucosa. By lowering acid levels, DGL helps mitigate mucosal damage and promotes ulcer healing, facilitating the restoration of mucosal integrity (El‐Saber Batiha et al. 2020; Singh et al. 2023).

Overall, specific plant‐derived compounds like DGL offer a targeted approach to reducing gastric acid secretion, providing symptomatic relief, and fostering an environment conducive to mucosal healing in individuals with peptic ulcers. By modulating proton pump activity, DGL represents a valuable adjunctive therapy in the management of peptic ulcers, offering benefits in both symptom management and ulcer healing.

The table of the North African medicinal plants indicates a significant prominence of families such as *Fabaceae*, *Asteraceae*, and *Lamiaceae*, which are frequently utilized for their gastroprotective properties. Notable compounds including flavonoids (e.g., quercetin and kaempferol), tannins, alkaloids, and essential oils are commonly identified, recognized for their anti‐inflammatory, antioxidant, and antimicrobial effects, all contributing to the treatment of peptic ulcers. The most utilized plant parts are the aerial parts, leaves, and roots, reflecting their high concentration of bioactive compounds.

## 6 Mechanisms of Action

**FIGURE 2:**
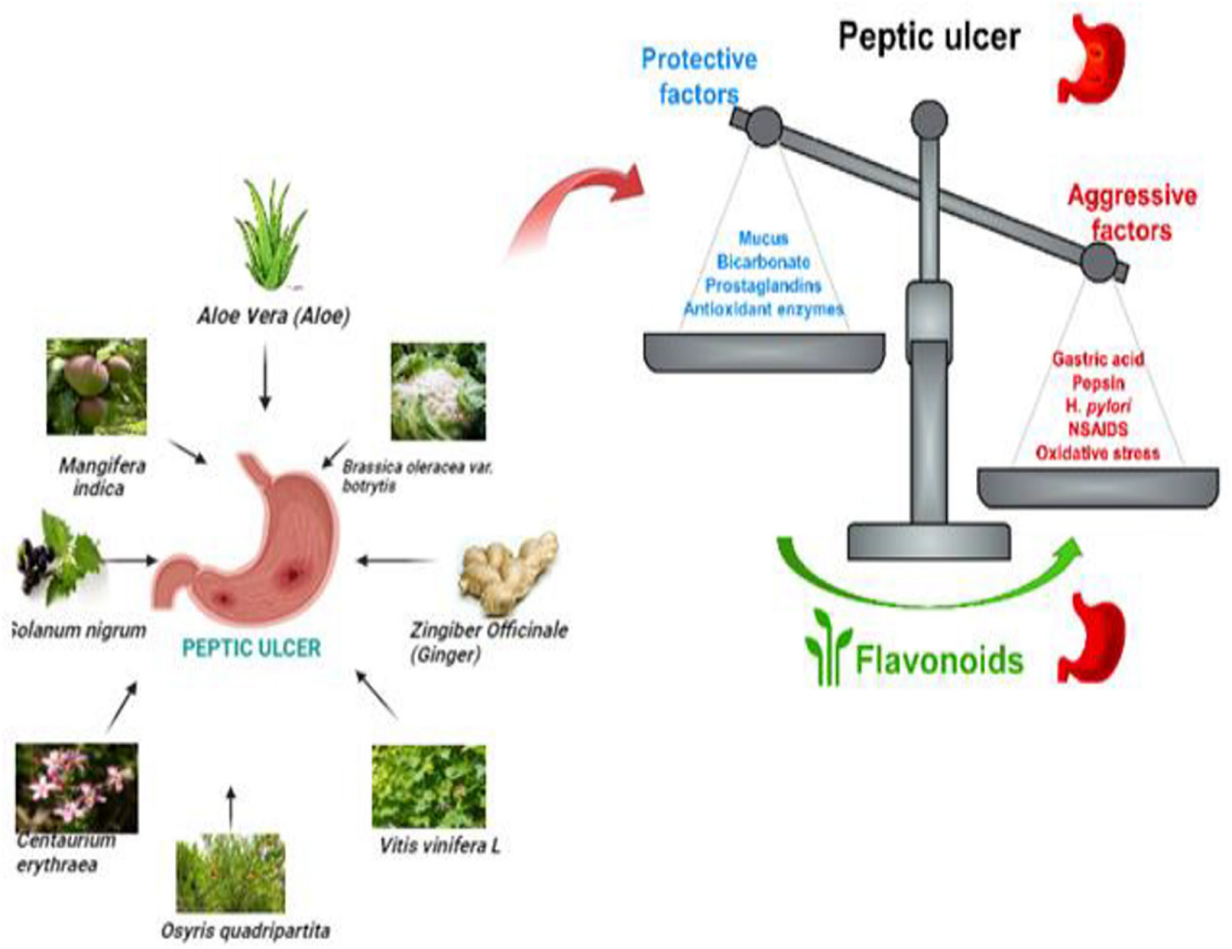
A figure summarizing how the mentioned medicinal plants affect peptic ulcers.

Gastric ulcers are commonly associated with *H. pylori* bacteria or overuse of nonsteroidal anti‐inflammatory drugs (NSAIDs) (Usta and Urganci 2015). Botanical compounds have demonstrated therapeutic potential in treating this gastrointestinal condition (Boakye‐Yiadom et al. 2021). Multiple plant‐derived metabolites including flavonoids, tannins, and terpenoids can modulate gastric acid output, reinforce the gastric mucosal barrier, and suppress *H. pylori* proliferation (Guevara and Cogdill 2020; Miri et al. 2022). These phytochemicals owe their gastroprotective effects to several mechanisms: inhibition of gastric acid secretion by blocking H+/K+‐ATPase action (Sachs et al. 1995), stimulation of mucus and bicarbonate output to reinforce the gastric mucus‐bicarbonate barrier (Zatorski 2017), elimination of *H. pylori* via antibacterial action (Guevara and Cogdill 2020; Miri et al. 2022), antioxidative action to counter inflammation and oxidative damage (Gündoğdu, Özbayer, and Kar 2023; Zaghlool et al. 2019), and enhancement of endogenous anti‐ulcer prostaglandins and nitric oxide while inhibiting pro‐ulcer leukotrienes and cytokines (Sánchez‐Mendoza et al. 2010). Through one or more of these mechanisms, plant bioactive can decrease ulcer index and increase curative ratio in chemically induced ulcer animal models (Yoo et al. 2022). The multi‐target synergistic effects of the phytochemical mixtures in medicinal plant extracts seemingly underlie their efficacy in protecting against gastric ulceration as well as enhancing healing of existing ulcers. Further pharmacodynamic and preclinical research is warranted to elucidate the most potent anti‐ulcer plant extracts and compounds for standardized botanical therapies (Yoo et al. 2022) (Figure 2).

## 7 Future Perspectives

The promising findings on North African medicinal plants for peptic ulcer treatment pave the way for exciting developments in both research and practical applications. Future studies should prioritize rigorous randomized controlled trials to establish the efficacy and safety of key plant extracts such as *Glycyrrhiza glabra*, *Matricaria chamomilla*, and *Nigella sativa* in human populations. Optimizing dosages, exploring potential synergies between plant compounds, and developing standardized extraction methods are crucial next steps. For instance, combining the antioxidant properties of *Olea europaea* with the mucoprotective effects of *Aloe vera* could yield enhanced therapeutic benefits. Long‐term studies on traditionally consumed plants like *Phoenix dactylifera* and *Opuntia ficus‐indica* may reveal their potential in ulcer prevention. Translating this research into practical applications could lead to a variety of product forms, including standardized herbal extracts in capsules, therapeutic teas blending multiple gastroprotective plants, topical gels containing *Aloe vera* or olive leaf extracts, and functional foods fortified with pomegranate or date palm extracts. The development of such products, alongside continued research, holds promise for more effective, natural approaches to managing peptic ulcers, potentially improving quality of life for millions affected by this condition.

## 8 Conclusion

In conclusion, this review highlights the gastroprotective efficacy and therapeutic potential of 54 North African medicinal plants for treating peptic ulcers. Notable plants such as licorice (*Glycyrrhiza glabra*), chamomile (*Matricaria chamomilla*), olive (*Olea europaea*), pomegranate (*Punica granatum*), *Aloe vera*, and black seed (*Nigella sativa*) demonstrated significant anti‐ulcer effects in preclinical studies, attributed to their bioactive compounds, including flavonoids, tannins, and terpenoids. These phytochemicals exhibit gastroprotective properties through various mechanisms, such as enhancing the gastric mucosal barrier, inhibiting acid secretion, displaying antioxidant and anti‐inflammatory effects, promoting ulcer healing, and combating *Helicobacter pylori* infection. The reviewed studies provide valuable insights into the potential of these medicinal plants as complementary or alternative therapies for peptic ulcer disease. However, it is important to acknowledge the limitations of the current evidence, which primarily consists of in vitro and animal studies, with limited human clinical trials. Additionally, variations in plant extraction methods, dosages, and study designs may impact the comparability and reproducibility of the findings. Future research should focus on conducting well‐designed, randomized, placebo‐controlled human trials to establish the safety and efficacy of these medicinal plants in preventing and treating peptic ulcers. Furthermore, investigations into the optimal dosage, formulation, and potential drug interactions are necessary to develop standardized therapeutic protocols. Elucidating the precise mechanisms of action and identifying the key bioactive compounds responsible for the gastroprotective effects will also aid in the development of targeted, evidence‐based interventions. While this review comprehensively covers the gastroprotective effects of North African medicinal plants, it may be beneficial to consider the potential for developing multi‐herb formulations that leverage synergistic effects between different plant compounds. Many traditional medicine systems utilize complex herbal formulas rather than single herbs. Given the diverse mechanisms of action highlighted for various plants in this review (antioxidant, anti‐inflammatory, mucus‐protective, etc.), there may be opportunities to create more potent and multi‐targeted gastroprotective preparations by strategically combining complementary herbs. This approach could potentially enhance efficacy while allowing for lower doses of individual herbs, potentially reducing side effects. Additionally, such formulations may better address the multifactorial nature of peptic ulcer disease. Overall, this review underscores the promising role of North African medicinal plants in the management of peptic ulcers and highlights the need for continued research to bridge the gap between traditional knowledge and modern therapeutic applications.

## Author Contributions

**Nezar Cherrada:** conceptualization (equal), formal analysis (equal), project administration (equal). **Ahmed Elkhalifa Chemsa:** resources (equal). **Noura Gheraissa:** validation (equal). **Ibtissam Laib:** investigation (equal). **Zakia Gueboudji:** software (equal). **Mohamed EL‐Shazly:** investigation (equal). **Abdelmalek Zaater:** validation (equal). **Asma Abid:** formal analysis (equal). **Sherouk Hussein Sweilam:** formal analysis (equal). **Talha Bin Emran:** visualization (equal). **Sadok Nani:** formal analysis (equal). **Bilal Benamor:** resources (equal). **Djilani Ghemam Amara:** resources (equal). **Ayomide Victor Atoki:** resources (equal). **Mohammed Messaoudi:** resources (equal).

## Ethics Statement

The authors have nothing to report.

## Conflicts of Interest

The authors declare no conflicts of interest.

## Data Availability

The data that support the findings of this study are included in the article. This encompasses all the original data generated during the research and any secondary data utilized in the analyses. For further inquiries or detailed information, the corresponding author can be contacted.
